# Integrative Analysis of Many Weighted Co-Expression Networks Using Tensor Computation

**DOI:** 10.1371/journal.pcbi.1001106

**Published:** 2011-06-16

**Authors:** Wenyuan Li, Chun-Chi Liu, Tong Zhang, Haifeng Li, Michael S. Waterman, Xianghong Jasmine Zhou

**Affiliations:** 1 Molecular and Computational Biology, Department of Biological Sciences, University of Southern California, Los Angeles, California, United States of America; 2 Department of Statistics, Rutgers University, New Brunswick, New Jersey, United States of America; ETH Zurich, Switzerland

## Abstract

The rapid accumulation of biological networks poses new challenges and calls for powerful integrative analysis tools. Most existing methods capable of simultaneously analyzing a large number of networks were primarily designed for unweighted networks, and cannot easily be extended to weighted networks. However, it is known that transforming weighted into unweighted networks by dichotomizing the edges of weighted networks with a threshold generally leads to information loss. We have developed a novel, tensor-based computational framework for mining recurrent heavy subgraphs in a large set of massive weighted networks. Specifically, we formulate the recurrent heavy subgraph identification problem as a heavy 3D subtensor discovery problem with sparse constraints. We describe an effective approach to solving this problem by designing a multi-stage, convex relaxation protocol, and a non-uniform edge sampling technique. We applied our method to 130 co-expression networks, and identified 11,394 recurrent heavy subgraphs, grouped into 2,810 families. We demonstrated that the identified subgraphs represent meaningful biological modules by validating against a large set of compiled biological knowledge bases. We also showed that the likelihood for a heavy subgraph to be meaningful increases significantly with its recurrence in multiple networks, highlighting the importance of the integrative approach to biological network analysis. Moreover, our approach based on weighted graphs detects many patterns that would be overlooked using unweighted graphs. In addition, we identified a large number of modules that occur predominately under specific phenotypes. This analysis resulted in a genome-wide mapping of gene network modules onto the phenome. Finally, by comparing module activities across many datasets, we discovered high-order dynamic cooperativeness in protein complex networks and transcriptional regulatory networks.

## Author Summary

To study complex cellular networks, we need to consider their dynamic topologies under many different experimental or physiological conditions. Integrative analysis over large numbers of massive biological networks thus emerges as a new challenge in data mining. Recently, we and others have proposed several algorithms for recurrent pattern mining across many () biological networks (with the main focus on unweighted networks). However, thus far no algorithms have been specifically designed to mine recurrent patterns across a large collection of weighted massive networks. In this paper, we propose a computational framework to identify recurrent heavy subgraphs from many weighted large networks. By applying our method to 130 co-expression networks, we identified an atlas of modules that are highly likely to represent functional modules, transcriptional modules, and protein complexes. Many of these modules would be overlooked with unweighted networks analysis. Furthermore, many of the identified modules constituted signatures of specific phenotypes. Finally, we demonstrated that our results facilitate the study of high-order dynamic coordination in protein complex networks and transcriptional regulatory networks.

## Introduction

The advancement of high-throughput technology has resulted in the accumulation of a wealth of data on biological networks. Co-expression networks, protein interaction networks, metabolic networks, genetic interaction networks, and transcription regulatory networks are continuously being generated for a wide range of organisms under various conditions. Thanks to this great opportunity, network biology is rapidly emerging as a discipline in its own right [1], [2]. Thus far, most computational methods have focused on the analysis of individual biological networks, but in many cases a single network is insufficient to discover patterns with multiple facets and subtle signals. There is an urgent need for methods supporting the integrative analysis of ***multiple*** biological networks. The analysis of multiple networks can be classified into two categories: (1) those studying conservations and evolvements of multiple networks between different species [3]–[8], and (2) those identifying shared network modules or variations of modules across multiple networks of the same species but under different conditions [9]–[15]. The two types of problems face different challenges. Cross-species network comparisons are typically carried out on tens of networks, with the bottleneck being the graph isomorphism problem caused by the possible many-to-many ortholog mapping; while the network comparison within the same species deal with hundreds of networks simultaneously, and their principal challenge is the large search space. In this paper, we will focus on the latter problem.

The analysis of multiple networks from the same species under different conditions has recently been addressed by ourselves and others with a series of heuristic data mining algorithms [9]–[14]. While useful, these methods still face two major limitations. (1) The general strategy of their searching heuristics is a stepwise reduction of the large search space, where each step involves one or more arbitrary cutoffs in addition to the initial cutoff that transforms continuous measurements (e.g. expression correlations) into unweighted edges. The *ad hoc* nature of these cutoffs has been a major criticism directed at this body of work [9]–[13]. (2) The cited algorithms cannot be easily extended to weighted networks. Most graph-based approaches to analyzing multiple networks are restricted to unweighted networks, and weighted networks are often perceived as harder to analyze [16]. However, weighted networks are obviously more informative than their unweighted counterparts. Transforming weighted networks into unweighted networks by dichotomizing weighted edges with a threshold obviously leads to information loss [17], and if there is no reasonable way to choose the threshold, this loss cannot be controlled. This paper presents a new method of analyzing multiple networks that overcomes both of these issues.

Generally speaking, a network of 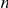 vertices can be represented as an 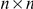 adjacency matrix 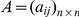, where each element 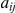 is the weight of the edge between vertices 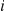 and 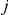. A number of numerical methods for matrix computation have been elegantly applied to network analysis, for example graph clustering [18]–[21] and pathway analysis [22], [23]. In light of these successful applications, we propose a tensor-based computational framework capable of analyzing many weighted and unweighted massive networks. Although tensor computation has been applied in the fields of psychometrics [24], [25], image processing and computer vision [26], [27], chemometrics [28], and social network analysis [29], [30], it has been explored only recently in large-scale data mining [31]–[35] and bioinformatics [36], [37].

**Figure 1:**
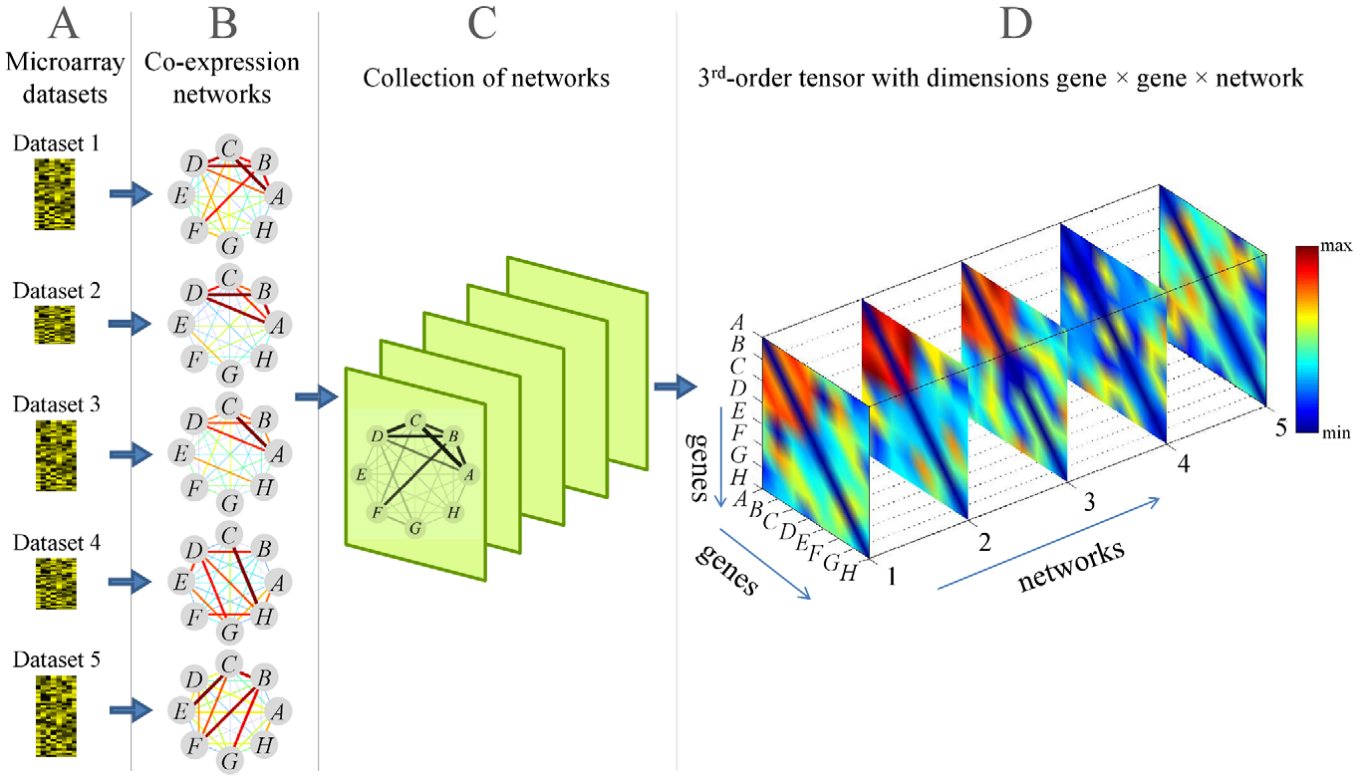
Illustration of the tensor representation for multiple networks and a recurrent heavy subgraph. (**A**) Microarray datasets are modeled as (**B**) a collection of co-expression networks; (**C**) These co-expression networks can be “stacked” together into (**D**) a third-order tensor such that each slice represents the adjacency matrix of one network. The weights of edges in the co-expression networks and their corresponding tensor elements are indicated by the color scale to the right of the figure. In (**D**), after reordering the tensor using the gene and network membership vectors, it becomes clear that the subtensor in the top-left corner of the tensor (formed by genes 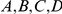 in networks 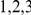) corresponds to a recurrent heavy subgraph.

Simply put, a tensor is a multi-dimensional array and a matrix is a 2nd-order tensor. Given 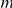 networks with the same 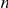 vertices but different topologies, we can represent the whole system as a 3rd-order tensor 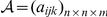 (see an example in Figure 1). Each element 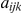 is the weight of the edge between vertices 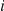 and 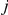 in the 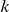 ^th^ network. By representing a set of networks in this fashion, we gain access to a wealth of numerical methods – in particular continuous optimization methods. In fact, reformulating discrete problems as continuous optimization problems is a long-standing tradition in graph theory. There have been many successful examples, such as using a Hopfield neural network for the traveling salesman problem [38] and applying the Motzkin–Straus theorem to solve the clique-finding problem [39]. Moreover, when a graph pattern mining problem is transformed into a continuous optimization problem, it becomes easy to incorporate constraints representing prior knowledge. Finally, advanced continuous optimization techniques require very few *ad hoc* parameters, in contrast with most heuristic graph algorithms.

In this paper, we develop a tensor-based computational framework to identify *recurrent heavy subgraphs* (RHSs) in multiple weighted networks. A *heavy subgraph* (HS) is a subset of heavily interconnected nodes in a single network. We define a RHS as a HS that appears in a subset of multiple networks. The nodes of a RHS are the same in each occurrence, but the edge weights may vary between networks. Although the discovery of heavy subgraphs in a single biological network can reveal functional and transcriptional modules [40]–[42], such results often contain false positives. Extending the search to a RHS is a promising way to enhance signal noise separation. Intuitively, any set of genes forming a RHS in multiple datasets generated under different conditions is more likely to represent a functional and transcriptional module than the genes in a single occurrence of a HS. We will use co-expression networks as examples due to their wide availability, but the tensor method described in this paper is applicable to any type of genome-wide networks.

The concept of a RHS can be explained using the language of tensors, as shown in Figure 1. Given 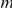 microarray datasets, we model each dataset with a co-expression network. Each node represents one gene, and each edge's weight is the estimated co-expression correlation of the two genes. We then “stack” the collection of co-expression networks into a three-dimensional array such that each slice represents the adjacency matrix of one network. This array is a third-order tensor 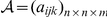 with dimensions gene
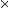
gene
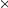
network. A RHS intuitively corresponds to a heavy region of the tensor (a heavy subtensor). The RHS can be found by reordering the tensor so that the heaviest subtensor moves toward the top-left corner. The subtensor in the top-left corner can then be expanded outwards from the left-top corner until the RHS reaches its optimal size.

We applied our tensor algorithm to 130 weighted co-expression networks derived from human microarray datasets. We identified an atlas of functional and transcriptional modules and validated them against a large set of biological knowledge bases including Gene Ontology annotations, KEGG pathways, 191 Encode genome-wide ChIP-seq profiles, and 109 Chip-chip datasets. The likelihood for a heavy subgraph to be biologically meaningful increases significantly with its recurrence, highlighting the importance of the integrative approach. Moreover, our approach based on weighted graphs detected many patterns that would have been overlooked if we were analyzing unweighted graphs. In addition, we identified many modules that occur predominately under a specific type of phenotypes. Thus, we were able to create a genome-wide mapping of gene network modules onto the phenome. Finally, based on module activities across multiple datasets, we used a high-order analysis approach to reveal the dynamic cooperativeness in protein complex networks and transcription regulatory networks.

## Methods

Given 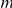 networks with the same 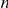 vertices but different topologies, we can represent the whole system as a 3rd-order tensor 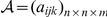. Each element 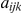 is the non-negative weight of the edge between vertices 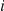 and 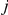 in the 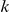 ^th^ network. Please note that 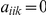 and 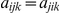 for any 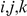, because we assume each network is undirected and without self-loops. Any *recurrent heavy subgraph* (RHS) can be described by two membership vectors: (i) the *gene membership vector* 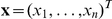, where 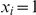 if gene 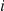 belongs to the RHS and 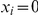 otherwise; and (ii) the *network membership vector* 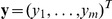, where 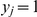 if the RHS appears in network 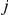 and 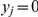 otherwise. The summed weight of all edges in the RHS is
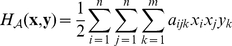
(1)Note that only the weights of edges 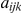 with 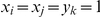 are counted in 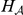. Thus, 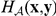 measures the “heaviness” of the RHS defined by 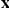 and 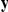. Discovering recurrent heavy subgraph can be formulated by a discrete combinatorial optimization problem: *among all RHSs of fixed size (* 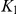 *member genes and* 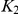 *member networks), we look for the heaviest.* More specifically, this is an integer programming problem of looking for the binary membership vectors 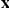 and 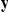 that jointly maximize 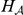 under the constraints 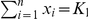 and 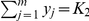. However, there are several major drawbacks to this discrete formulation. The first is *parameter dependence:* as with 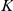-heaviest/densest subgraph problems, the size parameters 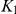 and 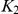 are hard for users to provide and control. The second is *high computational complexity:* the task is proved to be NP-hard (see Text S1) and therefore not solvable in reasonable time even for small datasets. As our own interest is pattern mining in a large set of massive networks, the discrete optimization problem is infeasible.

To address these two drawbacks, we instead solved a continuous optimization problem with the same objective by relaxing integer constraints to continuous constraints. That is, we looked for non-negative real vectors 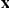 and 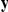 that jointly maximize 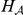. This optimization problem is formally expressed as follows:
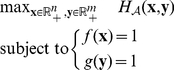
(2)where 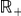 is a non-negative real space, and 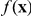 and 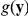 are vector norms. These equations define a tensor-based computational framework for the RHS identification problem. By solving Eq. (2), users can easily identify the top-ranking networks (after sorting the tensor by 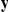) and top-ranking genes (after sorting each network by 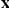) contributing to the objective function. After rearranging the networks in this manner, the heaviest RHS occupies a corner of the 3D tensor. We then mask this RHS with zeros and optimize Eq. (2) again to search for the next heaviest RHS.

Two major components of the framework described in Eq. (2) remain to be designed: (1) the vector norm constraints (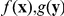), and (2) a protocol for maximizing 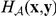. We explain our design choices below.

### Vector norm constraints

The choice of vector norms has a significant impact on the outcome of the optimization. The norm of a vector 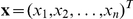 is typically defined in the form 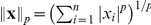, where 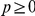. The symbol 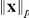, called the “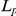-vector norm”, refers to this formula for the given value of 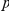. In general, the 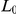 norm leads to sparse solutions where only a few components of the membership vectors are significantly different from zero [43]. The 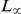 norm generally gives a “smooth” solution where the elements of the optimized vector are approximately equal. Details of these vector norms refer to Text S1.

A RHS is a subset of genes that are heavily connected to each other in as many networks as possible. These requirements can be encoded as follows. (1) *A subset of values in each gene membership vector should be significantly non-zero and close to each other, while the rest are close to zero*. To this end, we consider the mixed norm 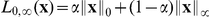 (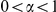) for 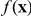. Since 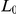 favors sparse vectors and 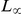 favors uniform vectors, a suitable choice of 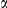 should yield vectors with a few similar, non-zero elements and many elements that are close to zero. In practice, we approximate 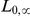 with the mixed norm 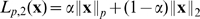, where 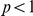. (2) *As many network membership values as possible should be non-zero and close to each other*. As discussed above, this is the typical outcome of optimization using the 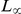 norm. In practice, we approximate 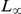 with 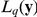 where 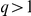 for 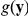. Therefore, the vector norms 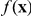 and 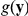 are specified as follows,
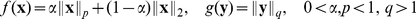
(3)We performed simulation studies to determine suitable values for the parameters 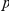, 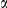, and 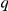 by applying our tensor method to collections of random weighted networks. In subsets of these networks, we randomly placed RHSs of varying size, occurrence, and heaviness. We then tried different combinations of 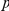, 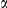, and 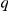, and adopted the combination (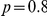, 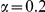, and 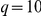) that led to the discovery of the most RHSs. More details on these simulations are provided in Text S1.

### Optimization by multi-stage convex relaxation

Since the vector norm 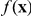 is non-convex, our tensor framework requires an optimization method that can deal with non-convex constraints. While the global optimum of a convex problem can be easily computed, the quality of the optimum discovered for a non-convex problem depends heavily on the numerical procedure. Standard numerical techniques such as gradient descent converge to a local minimum of the solution space, and different procedures often find different local minima. Considering the fact that most sparse constraints are non-convex, it is important to find a theoretically justified numerical procedure.

To design the optimization protocol, we use our previously developed framework known as Multi-Stage Convex Relaxation (MSCR) [43], [44]. MSCR has good numerical properties for non-convex optimization problems [43], [44]. In this context, concave duality is used to construct a sequence of convex relaxations that give increasingly accurate approximations to the original non-convex problem. We approximate the sparse constraint function 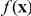 by the convex function 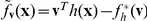, where 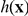 is a specific convex function 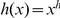 (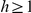) and 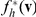 is the concave dual of the function 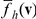 (defined as 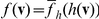). In practice, 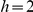 is an effective choice as the convex upperbound of 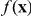. The vector 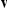 contains coefficients that will be automatically generated during the optimization process. After each optimization, the new coefficient vector 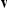 yields a convex function 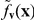 that more closely approximates the original non-convex function 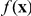.

The solution of our tensor formulation Eq. (2) is a stationary point of the following regularized optimization problem:
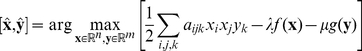
(4)where 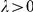 and 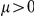 are Lagrange multipliers. By exploiting the concave duality of 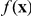, we can substitute 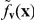 for 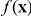. Therefore, Eq. (4) can be rewritten as
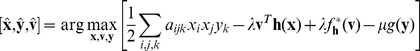
(5)


We solve Eq. (5) by repeatedly applying the following two steps:

- First, optimize 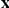 and 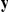 while holding 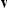 fixed.
- Second, optimize 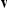 with 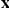 and 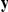 fixed. This problem has a closed form solution (for details, see Text S1).

The following box (see Box 1) presents our two-stage protocol to solve the regularized form of Eq. (2). The procedure can be regarded as a generalization of concave-convex programming [45], which takes 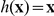. By repeatedly refining the parameters in 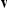, we can obtain better and better convex relaxations leading to a solution superior to that of the initial convex relaxation with 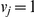. The initial values of 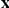 and 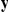 could be uniform, randomly chosen, or taken from prior knowledge. In practice, by choosing an appropriate solver for Step 1, the complexity of MSCR is linear with respect to the total number of edges in the tensor.

**Box 1. The Procedure of the Multi-Stage Convex Relaxation Method**

Inputs: tensor 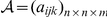, initial values 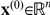 and 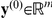.

Outputs: the gene membership vector 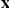 and network membership vector 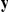

Initialize 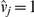.

Repeat the following two steps (referred to as a stage) until convergence:

- Step 1: let 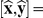 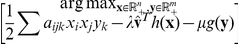.
- Step 2: let 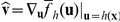.

For a detailed description of the optimization algorithm and procedure, see Text S1.

### Obtaining multiple recurrent heavy subgraphs

The RHSs can be intuitively obtained by including those genes and networks with large membership values. In practice, a pair of gene and network membership vectors 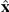 and 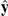, i.e., the solution of Eq. (2), can result in multiple RHSs whose “heaviness” is greater than a specified value (i.e., 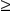 a threshold). Here, *the “heaviness” of a RHS is defined as the average weight of all edges in the RHS.*

**Figure 2:**
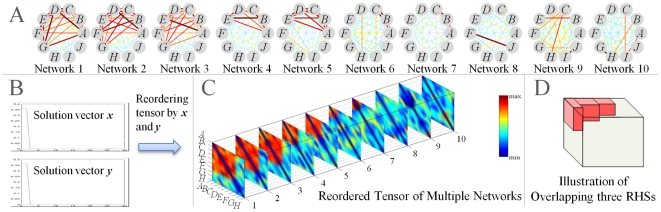
Illustration of an RHS family and its tower-like structure in 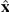 and 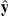. (**A**) Ten networks of 10 genes {A,B,C,D,E,F,G,H,I,J}, where the edge weight is associated with the color scale shown in (**C**); (**B**) The optimal membership vectors 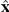 and 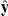 obtained by performing MSCR. Their significant components are ranked as follows: 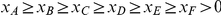, and 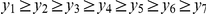; (**C**) The tensor of networks and genes arranged in decreasing order of the elements in 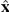 and 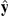. Three RHSs are discovered: the first RHS recurs in networks {1,2,3,4,5,6,7} with member genes {A,B,C}; the second recurs in networks {1,2,3,4,5} with member genes {A,B,C,D,E}; and the third recurs in networks {1,2,3} with member genes {A,B,C,D,E,F,G}; (**D**) A more intuitive illustration of three three overlapping RHSs, which form a tower-like structure.

In particular, the genes and networks are sorted in decreasing order of their membership values in 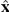 and 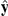. As illustrated by the example in Figure 2A–C, the more top-ranking genes are included in the RHS, the less networks the RHS recurs in; and vice versa. Such overlapping structure is like a tower as shown in Figure 2D. We refer to a group of overlapping RHSs that is obtained from the same pair of 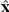 and 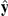 as a *RHS family*. To compress the redundant information, we build the representative RHSs for a RHS family as following: (1) if a RHS family contains multiple RHSs, the representatives are its two “extreme” RHSs: the RHS with the minimal number of genes (e.g., 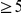) and as maximal recurrence as possible, and the RHS with the minimal number of networks (e.g., 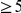) and as maximal number of genes as possible; (2) if a RHS family has only one RHS, it is the representative RHS.

After discovering the representative RHSs in this manner, we can mask their edges in the networks where they recur with zero weights and optimize Eq. (2) again to search for the next heaviest RHS. The source code of the algorithm is available at our Supplementary Website http://zhoulab.usc.edu/tensor/. This software is implemented by ANSI C and can be readily compiled and used in both Windows and Unix platforms.

### Non-uniform sampling for fast computation

Even though the MSCR method is efficient, its computation time can still be long for large sets of networks with many edges. In such cases, edge sampling can provide an efficient approximation to many graph problems [46], [47]. From the perspective of matrix or tensor computation, such sampling methods can be also viewed as matrix/tensor sparsification [48]. As RHS patterns predominately contain edges with large weights, we designed a non-uniform sampling method that preferentially selects edges with large weights. Specifically, each edge 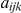 is sampled with probability 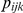:
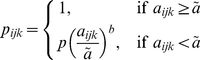
(6)where 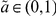, 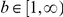 and 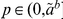 are constants that control the number of sampled edges. Note that Eq. (6) *always* samples edges with weights 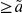. It selects an edge of weight 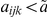 with probability 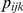 proportional to the 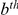 power of the weight. We choose 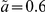, 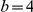, and 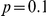 as a reasonable tradeoff between computational efficiency and the quality of the sampled tensor.

To correct the bias caused by this sampling method, the weight of each edge is corrected by its relative probability: 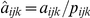. The expected weight of the sampled network, 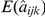, is therefore equal to the weight of the original network. However, in practice, when the adjusted edge weight 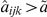 (but the original edge weight 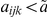), we enforced it to be 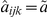 for avoiding too large edge weights. The overall edge sampling procedure adopts the simple random-sampling based single-pass sparsification procedure introduced in [48]. Details of the edge sampling procedure is provided in Text S1. After edge sampling, the procedure described above will use the corrected tensor 
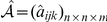
 instead of the original tensor 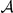.

### Data description and experimental setting

We selected every microarray dataset from NCBI's Gene Expression Omnibus that met the following criteria: all samples were of human origin; the dataset had at least 20 samples to guarantee robust estimates of the expression correlations; and the platform was either GPL91 (corresponding to Affymetrix HG-U95A), GPL96 (Affymetrix HG-U133A), GPL570 (HG-U133_Plus_2), or GPL571 (HG-U133A_2). We averaged expression values for probe that map to the same gene within a dataset. The 130 datasets that met these criteria on 28 January 2008 were used for the analysis described herein. Details are available at http://zhoulab.usc.edu/tensor/).

We applied our methods to these 130 microarray datasets. Each microarray dataset is modeled as a co-expression network wherein each node represents a unique gene and each edge weight represents the strength of co-expression of two genes. To determine the weights, we first compute the expression correlation between two genes as the leave-one-out Pearson correlation coefficient estimate [49]. The resulting correlation estimate is conservative and sensitive to similarities in the expression patterns, yet robust to single experimental outliers. To make the correlation estimates comparable across datasets, we then applied Fisher's z transform [50]. Given a correlation estimate 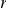, Fisher's transformation score is calculated as 
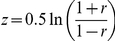
. Because we observed the distributions of 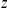-scores to vary from dataset to dataset, we standardized the 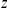-scores to enforce zero mean and unit variance in each dataset [11]. Then, the “normalized” correlations 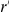 are obtained by inverting the 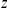-score. Finally, the absolute value of 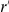 is used as the edge weight of co-expression networks. Details is provided in Text S1. In the other applications where networks contain negative edge weights, their edge weights can be transformed to be non-negative through translation, scaling or other transformation methods.

## Results

### Recurrent heavy subgraphs are likely to represent functional modules, protein complexes, and transcriptional modules

After applying our method to 130 microarray datasets generated under various experimental conditions, we identified 11,394 RHSs. Each RHS contains 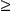
5 member genes, appears in 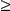
5 networks, and has a “heaviness” (defined as the average weight of its edges in networks where the RHS appears) 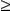
0.4. The average size of these patterns is 8.5 genes, and the average recurrence is 10.1 networks. The identified RHSs can be organized into 2,810 families with 4,327 representative RHSs, which we refer to in the following analysis. To assess the statistical significance of the identified RHSs, we applied our method to 130 random networks (each of which is generated from one of the 130 weighted networks by the edge randomization method) to identify RHSs with 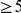 genes, 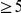 networks and “heaviness” 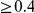. We repeated this process 100 times. None of RHSs were identified in any of the 100 times. When the minimum recurrence is 4 and other criteria remain unchanged, only 3 RHSs were found (Detail is provided in Text S1). To assess the biological significance of the identified RHSs, we evaluate the extent to which these RHSs represent functional modules, transcriptional regulatory modules, and protein complexes.

#### Functional module analysis

**Figure 3:**
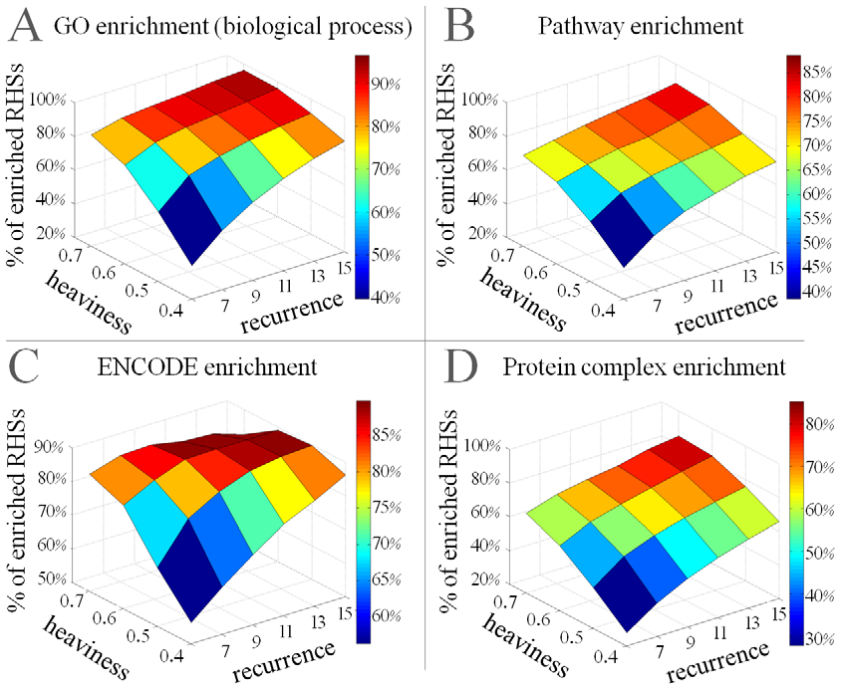
Evaluation of the functional, transcriptional, and protein complex homogeneity of RHSs with different recurrences and heaviness. Four types of databases are used: (**A**) Gene Ontology (GO) and (**B**) KEGG pathway databases for functional enrichment, (**C**) ENCODE database for transcriptional enrichment, and (**D**) CORUM database for protein complex enrichment. It can be seen that the percentage of potential functional, transcriptional, and protein complex modules increases with the heaviness and recurrence of the RHSs.

We evaluated the functional homogeneity of genes in an RHS using Gene Ontology and KEGG pathway information. For each RHS, we tested its enrichment for specific Gene Ontology (GO) biological process terms [51]. To ensure the specificity of GO terms, we filtered out those general terms associated with 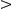
500 genes. If the member genes of an RHS are found to be significantly enriched in a GO term with a 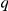-value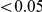 (the 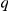-value is the hypergeometric 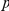-value after a False Discovery Rate multiple testing correction), we declare this RHS as functionally homogeneous. We found that 39.9% of the representative RHSs were functionally homogenous in this sense. In an ensemble of randomly generated RHSs having the same size distribution as our RHSs, only 1.2% of them were functionally homogenous. The functionally homogenous RHSs cover a wide range of biological processes: translational elongation, mitosis, cell cycle, RNA splicing, ribosome biogenesis, histone modification, chromosome localization, spindle checkpoint, posttranscriptional regulation, protein folding, etc. As shown in Figure 3A, not only RHSs with higher heaviness, but also those with high recurrences, are more likely to be functionally homogenous. For example, 40%/71%/90%/98% of patterns with 5/10/20/30 recurrences are functionally homogenous, as opposed to 4.30% of patterns with a single occurrence. This strong dependence highlights the importance of pursuing integrative analysis of *multiple* networks.

Similar results were achieved by using the KEGG database (http://www.genome.jp/kegg/) [52] to assess the association between RHS modules and known biological pathways. If the member genes of an RHS are significantly enriched in a pathway with a 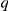-value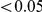, we declare the RHS to be pathway homogeneous. 38.6% of RHSs were pathway homogenous, compared to only 0.7% of randomly generated patterns (Figure 3B). Similarly, 39%/64%/78%/92% of patterns with 5/10/20/30 recurrences are functionally homogenous respectively, as opposed to 5.26% of patterns with a single occurrence.

**Figure 4:**
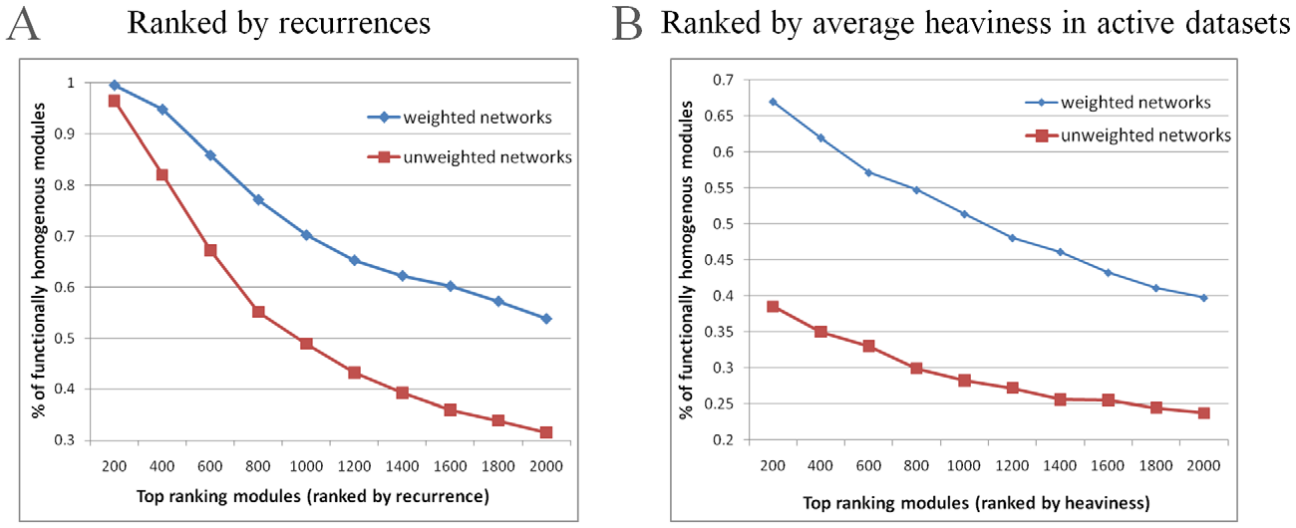
Comparison between weighted and unweighted network analysis. The weighted networks were transformed to unweighted networks by dichotomizing edges with an expression correlation cutoff of 0.6. The proposed tensor method was then applied to both weighted and unweighted networks. We compared rates of functional homogeneity detected in the top 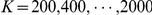 modules, ranked by (**A**) recurrences or (**B**) average heaviness in their datasets of occurrence. Weighted graph analysis consistently outperforms unweighted graph analysis.

It is important to note that our approach based on weighted networks discovers many patterns that would be overlooked if were using unweighted networks. For example, suppose we applied a commonly used expression correlation cutoff of 0.6 to dichotomize the edges, and a subnetwork density threshold of 0.6. In this case, 55.9% of our discovered RHSs are not discovered. To further avoid parameter biases in the comparison, we assess the functional homogeneity of the top-ranking 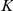 modules from both weighted and unweighted network analysis. The modules can be ranked by either their recurrences or their heaviness. In both ranking preferences, the weighted graph approach identifies a significantly higher percentage (up to 20%) of functionally homogenous modules than the unweighted graph approach (Figure 4), demonstrating the power and importance of weighted graph analysis.

#### Transcriptional module analysis

**Figure 5:**
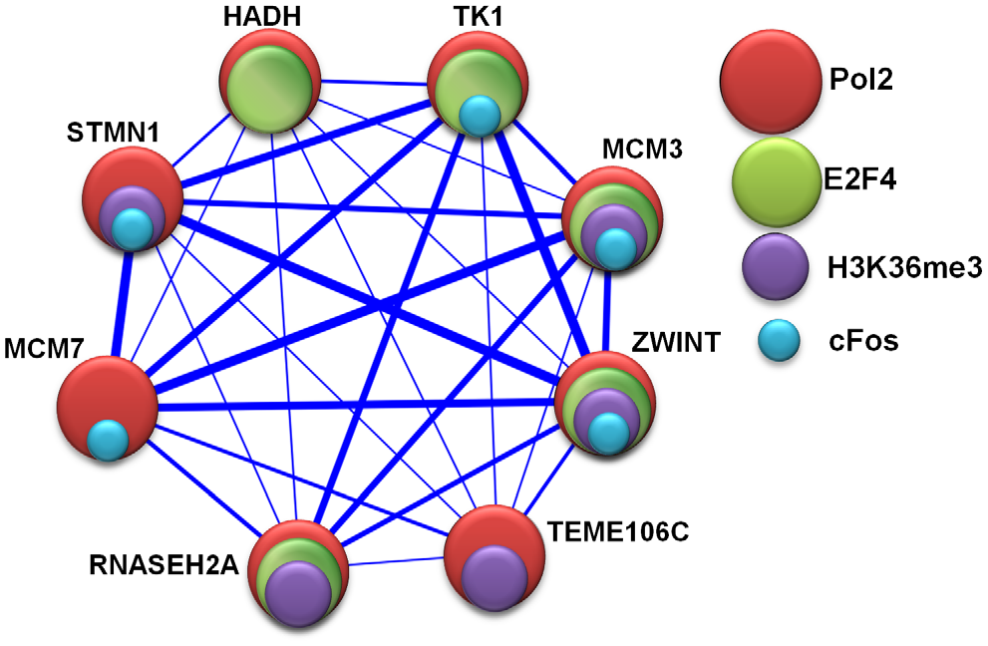
An 8-gene module is enriched in the binding of multiple regulatory factors. These regulatory factors are *Pol2* (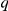-value = 1.73E-3), *H3K36me3* (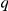-value = 5.54E-3), *E2F4* (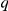-value = 1.65E-4), and *cFos* (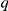-value = 2.68E-2). The module is active in 8 datasets, and its member genes are involved in DNA replication, 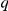-value = 2.15E-2).

Since genes in a RHS are strongly co-expressed in multiple datasets generated under different conditions, they are likely to represent a transcription module. To evaluate this possibility, we used the 191 ChIP-seq profiles generated by the Encyclopedia of DNA Elements (ENCODE) consortium [53]. This dataset includes the genome-wide binding of 40 transcription factors (TF), 9 histone modification marks, and 3 other markers (DNase, FAIRE, and DNA methylation) on 25 different cell lines. For a detailed description of the signal extraction procedure, see Text S1. These data provide a set of potential targets of regulatory factors that may or may not be active under a specific condition. However, if the member genes of a RHS are highly enriched in the targets for any regulatory factor, then that factor is likely to actively regulate the RHS under the given experimental conditions. In this case we consider the RHS module to be “transcriptional homogenous”. If we require an enrichment 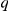-value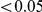, then 56.4% of the 4,327 RHSs with 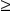
5 genes and 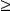
5 recurrences are transcription homogenous (compared to only 1.4% randomly produced RHSs). The percentage of transcription homogenous modules increases rapidly with heaviness and recurrence (Figure 3C). The five most frequently enriched regulators are *c-Myc* (enriched in 37.0% of RHSs), *Pol2* (38.2%), *DNase* (33.8%), *TAF II* (22.0%), and *E2F4* (20.9%). These results are not surprising. *c-Myc* and *E2F4* play important roles in cancer cells, and a large portion of our microarray data collection is related to cancer. *Pol2*, *DNase*, and *TAF II* are important for gene transcription in general. Remarkably, among the 4,327 modules, 2,108 (48.7%) are enriched in at least two factors, 1,926 (44.5%) in at least three factors; and 1,807 (41.8%) in at least four factors. These remarkable statistics highlight the combinatorial nature of transcriptional regulation. Figure 5 shows an example.

In addition, we collected 109 ChIP-chip experiments from published papers. Each experiment contains a set of targeting genes for a specific TF. After manually merging those TFs with synonymous names, this dataset involves 60 distinct TFs. Based on the above criteria, 24.8% of the 4,327 RHSs are enriched of at least one of these TFs (compared to 1.1% of randomly generated RHSs). Comparison between weighted and unweighted network analysis again showed that many transcription modules would be overlooked if using unweighted networks (details see Text S1).

#### Protein complex analysis

**Figure 6:**
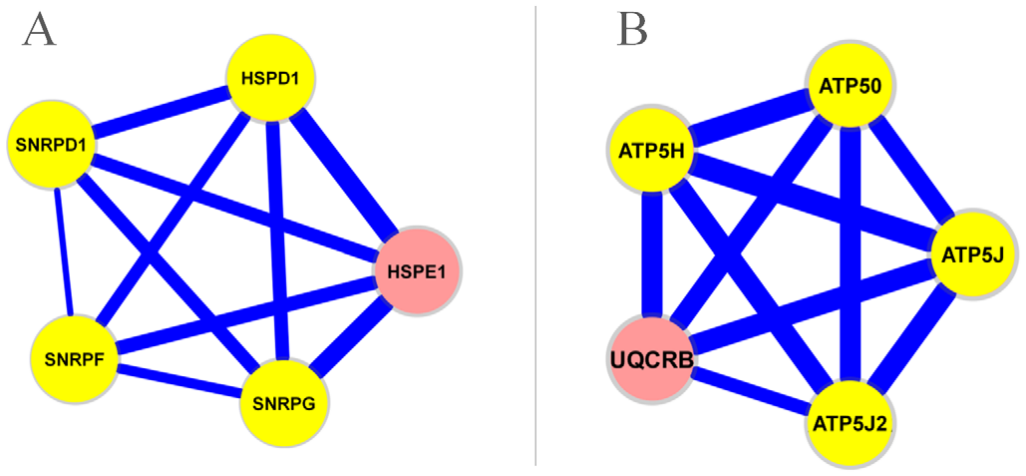
Two modules are enriched in protein complexes. The module in (**A**) is enriched in the *U2 snRNP* 17S protein complex (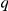-value = 9.9E-5) and the module in (**B**) is enriched in the *F1F0* ATPase protein complex (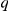-value = 1.8E-6). The members of the protein complexes are colored in yellow. The width of an edge is proportional to the average correlation of its genes in the datasets where the module occurs.

We applied our method to the Comprehensive Resource of Mammalian protein complexes (CORUM) database (http://mips.helmholtz-muenchen.de/genre/proj/corum/) [54] (September 2009 version). 27.8% of RHSs are significantly enriched with a 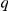-value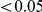 in genes belonging to a protein complex compared to only 0.16% of randomly generated patterns. The protein complexes are diverse and have a variety of functions. For example, a series of modules covered different parts of large complexes such as ribosome (both the small 40 s unit and the large 60 s unit), proteasome (the 20 s core unit and the 19 s regulatory unit), and splicesome. In addition, our modules represent a large number of small complexes; for example, multiple complexes involved in the cell cycle (e.g. *MCM* complex, *CDC2* complex, and *MCC* complex), the *CCT* micro-complex that serve as the chaperon for the folding of cytoskeleton proteins, the respiratory chain complex that is central to energy metabolism, and the *SMN* complex that plays an essential role in the assembly of *snRNPs*. Figure 6 illustrates two examples.

### Discovery of phenotype-specific modules

Our microarray data collection covers a wide range of phenotypic conditions, especially most of all, many different types of cancers (cancers accounts for 46% of the datasets). If an RHS is activated repeatedly *only* under one type of phenotypic condition, then it is likely to contribute specifically to the molecular basis of the phenotype. It is known that phenotypes are determined not only by genes, but also by the underlying structure of genetic networks. While traditional genetic studies have sought to associate single genes with a particular phenotypic trait, identifying phenotype-specific network modules has been a challenge of network biology. Below we show that a large number of the RHSs identified by our method are indeed phenotype-specific.

First, we determined the phenotypic context of a microarray dataset by mapping the Medical Subject Headings (MeSH) of its PubMed record to UMLS concepts. We used the MetaMap Transfer tool provided by the UMLS [55] for this purpose. UMLS is the largest available compendium of biomedical vocabularies, spanning approximately one million interrelated concepts. It includes diseases, treatments, and phenotypic concepts at several levels of resolution (molecules, cells, tissues, and whole organisms). We annotated each microarray dataset with matching UMLS concepts and all of their ancestor concepts. For each RHS, we evaluated phenotype specificity by computing the hypergeometric enrichment of specific UMLS concepts present in those datasets where the RHS occurs. If the 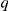-value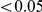, we consider the RHS module is significantly phenotype-specific. 5.62% of RHSs show phenotype-specific activation patterns, compared to 0.14% of randomly generated RHSs. The most frequently enriched phenotype concepts are related to cancer. For example, the most prevalent concepts are “Leukemia, Myelocytic, Acute” (enriched in 1.8% of modules) and “Neoplasms, Neuroepithelial” (1.3%). Among non-cancer concepts, the most frequent are “Respiratory Tract Diseases” (enriched in 0.2% of modules), “Bone Marrow Diseases” (0.2%) and “Lung diseases” (0.1%). Below we illustrate two examples of phenotype-specific modules.

**Figure 7:**
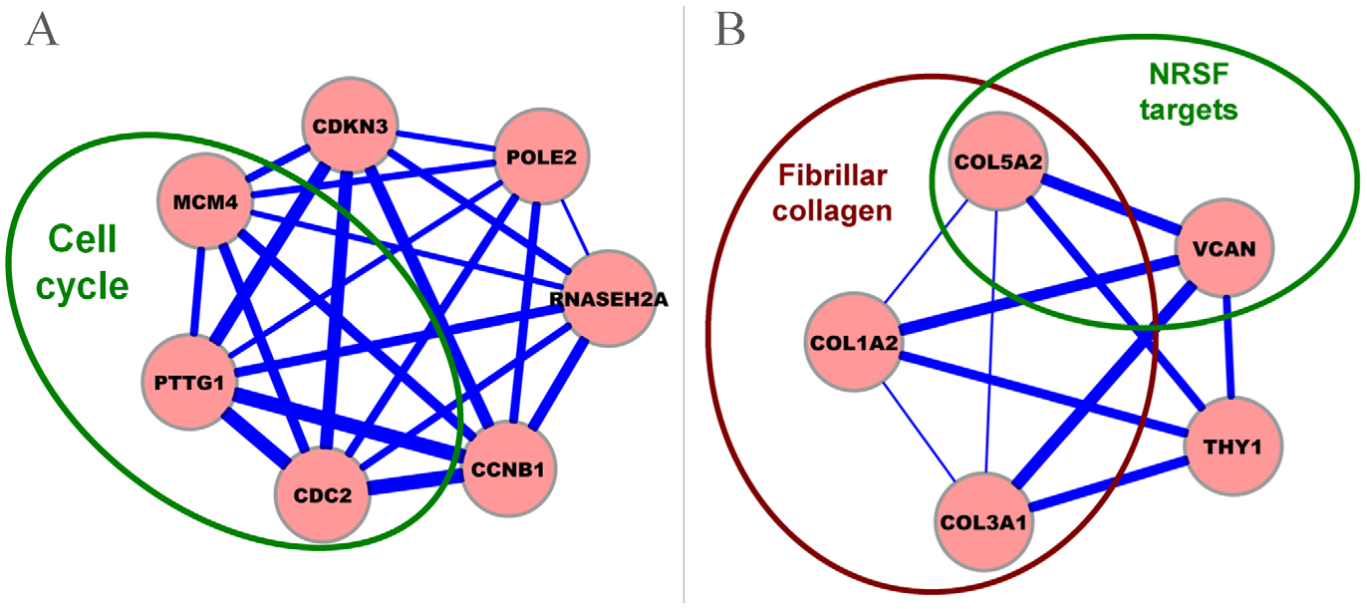
Examples of phenotype-specific modules associated with (A) Glioma and (B) muscle. The width of an edge is proportional to the average correlation of its genes in the datasets where the module occurs.

Figure 7A shows a 7-gene module (*CCNB1*, *POLE2*, *CDC2*, *PTTG1*, *RNASEH2A*, *CDKN3*, *MCM4*) that is active in 21 datasets. Twelve of the 21 datasets are related to cancer, and three relate to the study of Glioma (GDS1975, GDS1815, GDS1962) (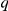-value = 0.075). Interestingly, four out of the seven genes are known to be associated with Glioma. *CCNB1* and *CDC2* play important roles in the proliferation of Glioma cells [56], the expression of *PTTG1* is correlated with poor prognosis in Glioma patients [57], and aberrant splicing of *CDKN3* increases proliferation and migration in Glioma cells [58]. This knowledge confirms our prediction of the module's strong association with Glioma. This module is enriched in genes from the cell cycle pathway (*CCNB1*, *CDC2*, *PTTG1*, and *MCM4*; 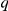-value = 1.08E-3).

Figure 7B shows a 5-gene module(*COL3A1*, *COL1A2*, *COL5A2*, *VCAN*, *THY1*) that is active in 22 datasets. Four of these datasets study expression in muscle tissue (GDS914, GDS563, GDS268, GDS2055) (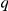-value = 0.03). This module contains 3 genes (*COL3A1*, *COL1A2*, *COL5A2*) annotated with fibrillar collagen (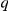-value = 8.41E-4), a major component of muscle (especially cardiac skeleton). Furthermore, *COL1A2* and *VCAN* are targeted by neuron-restrictive silencer factor (*NRSF*). Notably, [59] has reported that the *NRSF* maintains normal cardiac structure and function and regulates the fetal cardiac gene program. In addition, *VCAN* plays a role in conditions such as wound healing and tissue remodeling in the infracted heart [60]. Four out of five genes in the module are associated with muscle, providing strong evidence for phenotype specificity.

### High-order cooperativity and regulation in protein complex networks and transcription regulatory networks

The discovery of RHS modules spanning a variety of experimental or disease conditions enabled us to investigate high-order coordination among those modules. We applied our previously proposed *second-order analysis* to study cooperativity among the protein complexes[49]. We define the first-order expression analysis as the extraction of patterns from one microarray data set, and the second-order expression analysis as a study of the correlated occurrences of those patterns (e.g. heavy subgraph recurrence) across multiple data sets. Here, for each identified RHS, we constructed a vector of length 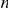 storing its heaviness factors in the 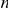 data sets. This vector is a profile of the module's first-order average expression correlations, and can be interpreted as the activity profile of the module in different datasets. To quantify the cooperativity between two modules, we calculated the correlation between their first-order expression correlation profiles. It is defined as the *second-order expression correlation* of the two modules.

**Figure 8:**
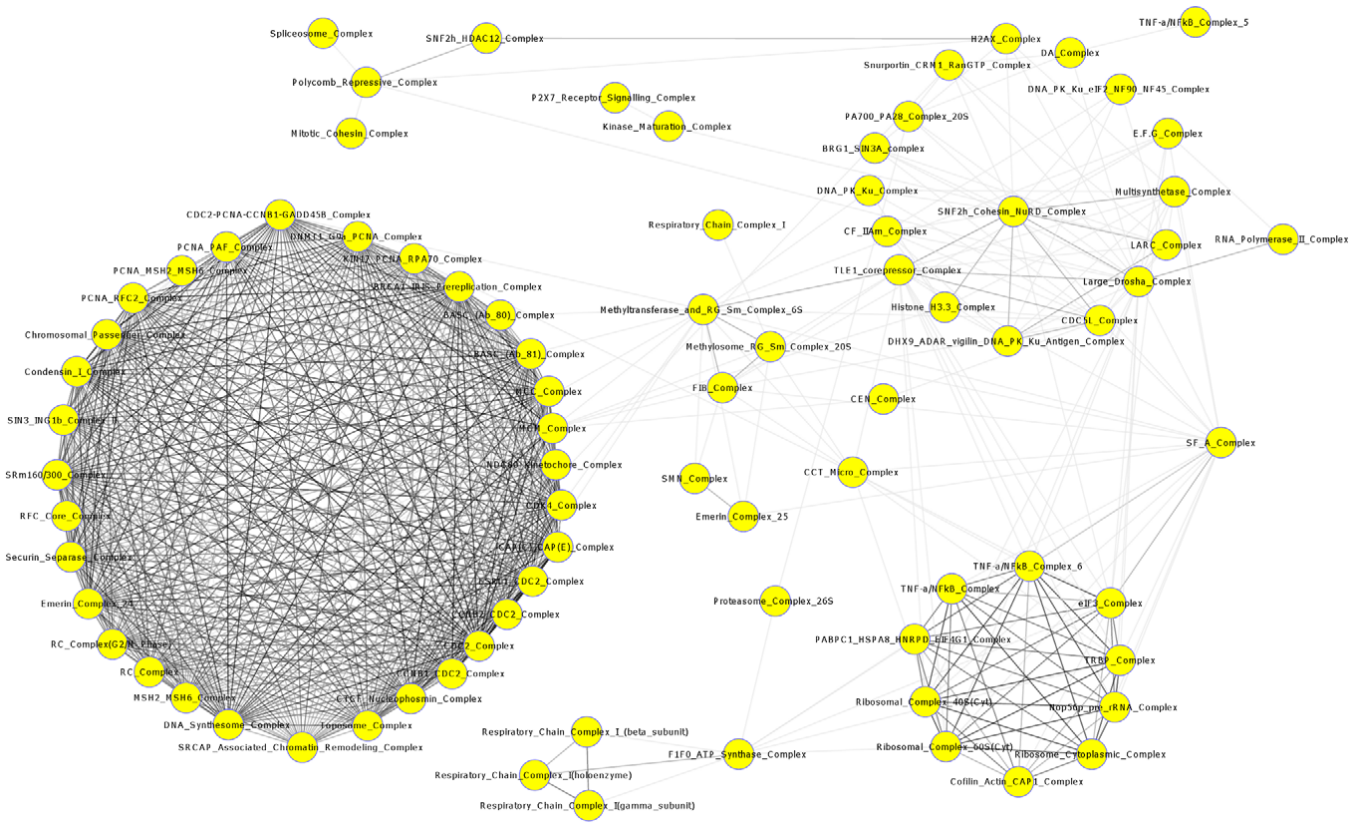
The protein complex cooperativity network. Nodes represent protein complexes, and edges represent high (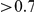) second-order correlation between pairs. The second-order correlation quantifies the cooperativity of activities of the two RHSs modules across different datasets. The darker the color of the edges, the stronger the second-order correlation.

Figure 8 shows a cooperativity map of all protein complexes represented by the RHSs that have high (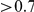) second-order correlations with at least one other protein complexes. The most striking feature of this map is a large and very heavily interconnected subnetwork of 32 complexes, all involved in the cell cycle. Within this subnetwork, 17 complexes (including *CDC2*_Complex, *CCNB2_CDC2*_Complex, *CDK4*_Complex, Chromosomal_Passenger_Complex, and Emerin_Complex_24) form a tight core wherein each complex has strong second-order correlations (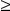
0.95) with all others in the core. This structure highlights the strict transcription regulation of cell cycle processes. Two other prominent dense subnetworks contain protein complexes involved in the respiratory chain and those in translation (e.g. the ribosomal complex, the *NOP56* associated pre-RNA complex, and the *TRBP* complex associated with miRNA dicing). Another loosely coupled subnetwork contains protein complexes mainly involved in transcription and post-transcriptional modification, including the participating members of *CDC5L* complex (pre-mRNA splicing), *CF IIAm* complex (pre-mRNA cleavage), *SNF2h*-cohesion-NuRD complex (chromatin remodeling), DA complex (transcription activation), and the large drosha complex (primary miRNA processing), revealing the tight coupling between transcription and post-transcriptional processes. Numerous protein complexes (e.g. *CEN* complex, *FIB*-associated complex, and *CCT* complex) connect these dominant subnetworks or supercomplexes into an integrated network. Thus, our approach not only provides a comprehensive catalogue of modules that are likely to represent protein complexes, but also the very first systematic view of how protein complexes dynamically coordinate to carry out major cellular functions. That is, by integrating data generated under a variety of conditions, we have gained a glimpse into the activity organization chart of the proteome.

**Figure 9:**
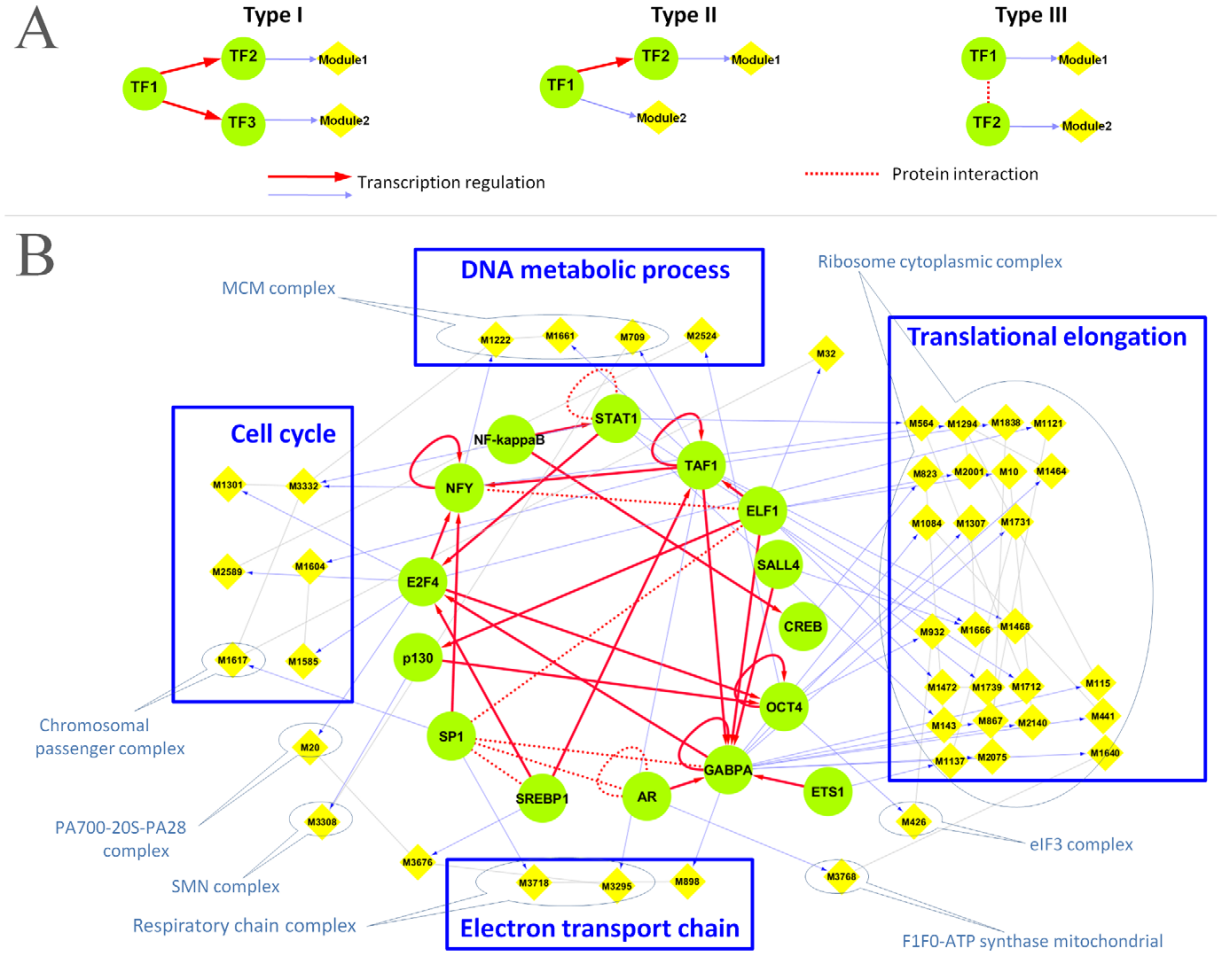
Reconstruction of transcriptional regulatory networks. (**A**) Three types of possible transcription networks that could explain a second-order correlation between two transcriptional modules. Given two modules controlled by two transcription factors, TF1 and TF2, respectively, the coactivation of the two modules implies cooperativity between TF1 and TF2. This relationship may be caused by a type I network in which the activities of TF1 and TF2 are controlled by common transcription factor(s) TF3; or a type II network, in which the activity of TF2 is controlled by TF1 or vice versa; or a type III network, in which TF1 and TF2 interact at the protein level. (**B**) A regulatory network reconstructed on the basis of the derived transcription networks. Green circles denote transcription factors, yellow boxes are transcription modules defined by RHSs (detailed information on these RHSs provided in Text S1), blue ovals denote protein complexes represented by the RHSs, and blue boxes highlight the biological processes in which the modules are involved.

The same principle can be applied to uncover the cooperativity among the transcription modules, thereby reconstructing transcriptional networks. The RHS discovery resulted in an atlas of transcription modules activated under different conditions. Each transcription module can be regulated by one or more transcription factors. Intuitively, if two transcription modules form or do not form two co-expression clusters always under the same set of conditions (that is, in the same data sets), it in fact suggests that their respective transcription factors are active or inactive simultaneously. The cooperativity between two sets of transcription factors can again be quantified using second-order expression correlation, since the the activity of a transcription factor can be assessed by the tightness of co-expression among the genes it regulates, i.e., the first-order profiles of the corresponding RHS module. We focus on the 57 transcription factors with enriched targets in our modules. Among these TFs, we identified 25 TF pairs, each of which regulate two distinct modules with second-order correlations greater than 0.7. We traced the potential sources of cooperativity in these pairs using genome-wide TF binding data and protein-protein interaction data [61]. Given two modules controlled respectively by transcription factors TF1 and TF2, which for simplicity are assumed to be individuals instead of sets of transcription factors, there are at least three types of possible direct causes of cooperativity between TF1 and TF2 (Figure 9A): the expressions of TF1 and TF2 are activated by a common transcription factor TF3 (a type I transcription network), or TF1 activates the expression of TF2 (a type II transcription network), or TF1 and TF2 interact at the protein level (a type III transcription network). In the special case where a module pair shares the majority of common genes, the cooperativity between TF1 and TF2 is known to be combinatorial control. Note that these three types of transcription networks are certainly only a few of the many possibilities.

We identified 33 transcription networks, among which 10 networks are of Type I, 19 are of Type II, and 4 are of Type III. These transcription networks interconnect to form a partial cellular regulatory network (Figure 9). Four networks are involved in the cell cycle: the Type I network involving *SREBP1* and *TAF1*/*E2F4*, the two Type II networks involving *STAT1* and *E2F4* as well as *SP1* and *NFYA*, and the Type III network involving *ELF1* and *SP1*. The roles of these networks are supported by the independent evidence of cooperative roles of those transcription factors reported in the literature [62]–[65]. Other transcription networks participate in translational elongation, rRNA processing, RNA splicing, DNA replication, DNA packaging, electron transport, etc. Notably, our reconstructed transcriptional regulatory network includes 35 modules that represent protein complexes, which provides a mechanistic explanation for the correlated activities of those protein complexes, as shown in Figure 8. For example, cooperativity between the chromosome passenger complex *CPC* and the *MCM* complex (see Figure 9B) can be attributed to the Type II networks between their regulators *E2F4* and *NFY*. This is consistent with previous evidences on the synergistic activities between the two transcription factors [66]. Strikingly, the protein complexes in the ribosome that participate in the translational elongation are regulated by a network of intertwined transcription networks. This highlights the regulatory complexity of the translation process, an impressive feat given that the TFs used in this study represent only a very small fraction of the TF repertoire.

## Discussion

We have developed a novel tensor-based approach to identify recurrent heavy subgraphs in many massive weighted networks. This is the first method suitable for pattern discovery in large databases of many weighted biological networks. We applied the method to 130 co-expression networks, and identified a large number of functional and transcriptional modules. We show that the likelihood for a heavy subgraph to be meaningful increases significantly with its recurrence in multiple networks, highlighting the importance of the integrative approach for network analysis. By analyzing databases of networks derived from a wide range of experimental conditions, we can also study the high-order dynamic coordination of modules, a task that can be hardly addressed using only a single network. In addition, the phenotype information associated with gene expression datasets provides opportunities to perform systematic genotype-phenotype mapping [14], [67]. Among our identified modules, many have been shown to be phenotype-specific. While weighted networks are often perceived as harder to analyze than their unweighted counterparts, we show that many patterns are overlooked if using the unweighted networks. Although currently unweighted networks (protein-protein interaction network, genetic interaction network, and metabolic network, etc.) still dominate biological studies, rapidly evolving genomics technology will soon be able to provide quantitative assessments of those interactions, thus resulting in accumulated weighted networks. Our method is well positioned to respond to the emerging challenges of network biology.

## Supporting Information

Text S1 The supplementary text to give the detailed supplementary information of the methods and results.
(1.25 MB PDF)
