# Supplementary material for: Integrative Analysis of Many Weighted Co-Expression Networks Using Tensor Computation
Source: PLoS Comput Biol. 2011 Jun 16;7(6):e1001106. doi: 10.1371/journal.pcbi.1001106 (PMC3116899; doi:10.1371/journal.pcbi.1001106)
Supplement: Text S1 — The supplementary text to give the detailed supplementary information of the methods and results. (1.25 MB PDF) [file pcbi.1001106.s001.pdf]

Supplementary Text S1

Integrative Analysis of Many Weighted Coexpression Networks Using Tensor Computation

Wenyuan Li
Molecular and Computational Biology, University of Southern California
Los Angeles, CA 90089, USA

Chun-Chi Liu
Molecular and Computational Biology, University of Southern California
Los Angeles, CA 90089, USA

Tong Zhang
Department of Statistics, Rutgers University, NJ, USA

Haifeng Li
Molecular and Computational Biology, University of Southern California
Los Angeles, CA 90089, USA

Michael S. Waterman
Molecular and Computational Biology, University of Southern California
Los Angeles, CA 90089, USA

Xianghong Jasmine Zhou
Molecular and Computational Biology, University of Southern California
Los Angeles, CA 90089, USA
xjzhou@usc.edu

Contents

S1 NP-hardness of the Heaviest (K_1, K_2)-RHS Problem **S3**

S2 Details of vector norms **S4**

S3 Details of multi-stage convex relaxation method **S4**
S3.1 Concave duality S5
S3.2 Multi-stage convex relaxation S5

S4 Simulation study **S7**

S5 The edge sampling procedure and its theoretical analysis **S8**
S5.1 Sampling procedure S8
S5.2 Theoretical analysis S8

S6 Details of computing platform and running time **S9**

S7 Details of normalization procedure of pairwise gene co-expression correlations **S10**

S8 Random network generation and comparison with real networks **S11**

S9 Signal extraction procedure of ENCODE data **S11**

S10 Comparisons with unweighted networks **S12**

S11 Detailed information of modules in the regulatory network reconstructed in Figure 9 of the manuscript **S12**

S1 NP-hardness of the Heaviest (K_1, K_2)-RHS Problem

Given a set of m undirected graphs $G_1, \ldots, G_m$ with the same n vertices V but different topologies (and without self-loops), i.e., $\mathcal{G} = \{G_1(V, E_1), \ldots, G_m(V, E_m)\}$ with $V = \{v_1, \ldots, v_n\}$ and non-negative weights a_{ijk} for edges $(v_i, v_j) \in E_k$ in the kth graph, the (K_1, K_2)-RHS problem is formally defined as follows,

Problem S1.1. *Given $\mathcal{G}$, the (K_1, K_2)-*Recurrent Heavy Subgraph (RHS) *problem is to determine a subset $S_V \in V$ of K_1 vertices and a subset $S_G \in \mathcal{G}$ of K_2 graphs such that the total sum of edge weights of the subgraphs induced by S_V in each graph of S_G is maximized. A straightforward cubic 0-1 formulation of (K_1, K_2)-RHS is*

$$\begin{array}{ll} \max & \frac{1}{2}\sum_{i=1}^{n}\sum_{j=1}^{n}\sum_{k=1}^{m} a_{ijk}x_i x_j y_k \\ \textit{subject to} & \begin{cases} \sum_{i=1}^{n} x_i = K_1 \\ \sum_{i=1}^{m} y_j = K_2 \\ x_i \in \{0,1\} \quad & \textit{for any } 1 \leqslant i \leqslant n \\ y_j \in \{0,1\} \quad & \textit{for any } 1 \leqslant j \leqslant m \end{cases} \end{array} \tag{1}$$

Next we prove the NP-hardness of this problem.

Theorem S1.2. *The (K_1, K_2)-recurrent heavy subgraph problem is NP-hard.*

Proof. We can reduce the well-known NP-complete K-clique problem [1] (i.e., *is there a clique with K vertices in a graph?*) to this problem, and therefore prove its NP-hardness.

Let $G(V, E)$ be an undirected unweighted graph without self-loops. We can copy this graph m times to generate a graph set consisting of the m graphs $\mathcal{G} = \{G_1(V, E), \ldots, G_m(V, E)\}$ in which all graphs have the same vertex set V and edge set E. Then the question of "*is there a clique with K vertices in G?*" can be answered by solving the (K, K_2)-RHS problem in the set of m graphs $\mathcal{G}$, because we can easily claim:

- *If the heaviest (K, K_2)-RHS found in the graph set $\mathcal{G}$ is a clique with K vertices recurring in the K_2 graphs, then there exist a clique with K vertices in G.* This claim is obvious and straightforward.
- *If the heaviest (K, K_2)-RHS found in the graph set $\mathcal{G}$ is not a clique with K vertices recurring in the K_2 graphs, then a clique with K vertices does not exist in G.* This claim can be proved by contradiction: supposing there exists a clique with K vertices in G, since all graphs G_i in the graph set $\mathcal{G}$ were copied from G, this K-clique in G must also exist at least K_2 graphs of the graph set $\mathcal{G}$. Among all (K, K_2)-RHSs in m unweighted graphs, the K-cliques recurring in K_2 graphs must be the (K, K_2)-RHS having the largest total sum of edge weights. This K-clique recurring in K_2 graphs must be the solution of the heaviest (K, K_2)-RHS. So it contradicts with the statement "the heaviest K, K_2-RHS found is not a clique with K vertices recurring in the K_2 graphs".

Since the K-clique problem is NP-complete, the *(K_1, K_2)-RHS* problem is NP-hard. □

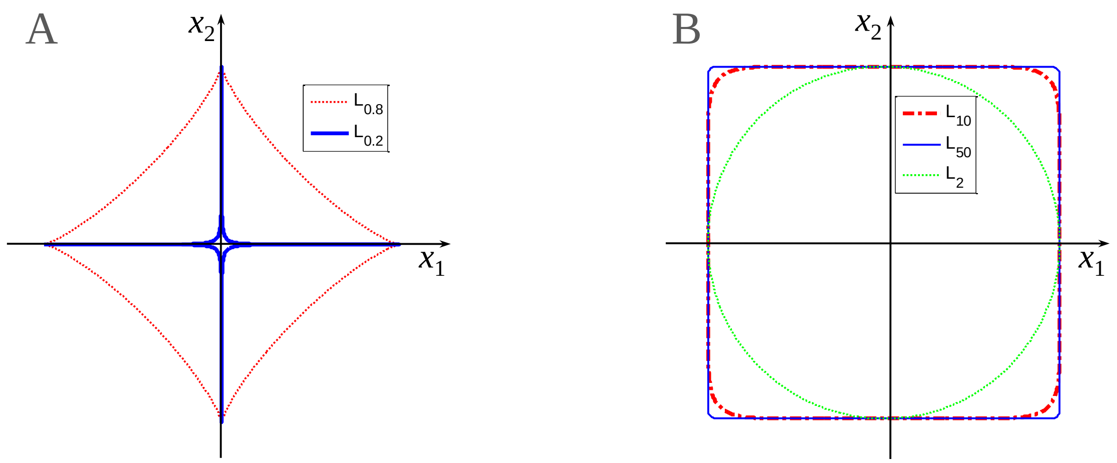

Figure S1. Two-dimensional contour plots of the vector norm constraints. (A) contour of the vector norm L_p $(0 < p < 1)$. When $p \to 0$, $L_p \to L_0$ and therefore L_p can make $\mathbf{x} = (x_1, x_2)^T$ sparser. (B) Contour of the vector norm L_p $(p > 1)$. When $p \to \infty$, $L_p \to L_\infty$ and therefore L_p can make $\mathbf{x} = (x_1, x_2)^T$ more even)

S2 Details of vector norms

The L_p $(p > 0)$ norm of a vector $\mathbf{x} \in \mathbb{R}^{n\times 1}$ is defined as $\|\mathbf{x}\|_p = (\sum_{i=1}^{n} |x_i|^p)^{1/p}$. Two extreme cases of L_p norm is zero norm $L_0 = \text{card}\{x_i | x_i \neq 0\}$, where card is the set cardinality (i.e., the number of non-zero elements of $\mathbf{x}$), and infinity norm (or maximum norm) $L_\infty = \max\{x_1, \ldots, x_n\}$. When the L_p vector norm is used as optimization's constraint, the set of all vectors with norm 1 (i.e., $L_p(\mathbf{x}) = 1$) defines the feasible region, whose two-dimensional case is shown in Figure S1. It can be observed that the closer p is to zero, the sparser $\mathbf{x}$ is; while the closer p is to ∞, the smoother or more even $\mathbf{x}$ is. In practice, L_p with $p < 1$ is often used to approximate L_0, and L_p with $p \geqslant 2$ is often used to approximate L_∞.

S3 Details of multi-stage convex relaxation method

We use our previously developed framework known as Multi-Stage Convex Relaxation (MSCR) [1] to design the optimization protocol. In this context, concave duality will be used to construct a sequence of convex relaxations that give increasingly accurate approximations to the original non-convex problem. We approximate the sparse constraint function $f(\mathbf{x})$ by the convex function $\widetilde{f}_{\mathbf{v}}(\mathbf{x}) = \mathbf{v}^T h(\mathbf{x}) - f_h^*(\mathbf{v})$, where $h(\mathbf{x})$ is a specific convex function $h(x) = x^h$ $(h \geqslant 1)$ and $f_h^*(\mathbf{v})$ is the concave dual of the function $\overline{f}_h(\mathbf{v})$ (defined as $f(\mathbf{v}) = \overline{f}_h(h(\mathbf{v}))$. The vector $\mathbf{v}$ contains coefficients that will be automatically generated during the optimization process. After each optimization, the new coefficient vector $\mathbf{v}$ yields a convex function $\widetilde{f}_{\mathbf{v}}(\mathbf{x})$ that more closely approximates the original non-convex function $f(\mathbf{x})$.

[1]The NP-completeness proof of the K-clique problem can be found in textbooks introducing "algorithms and complexity" and lectures' exercises, e.g., `http://people.bath.ac.uk/masnnv/Teaching/AAlg10Sol.pdf`.

S3.1 Concave duality

Given a continuous regularization function $f(\mathbf{x})$ which may be non-convex, we are interested in rewriting it using concave duality. Detail refer to [1]. Let $\mathbf{h}(\mathbf{x}) : \mathbb{R}^n \to \Omega \subset \mathbb{R}^n$ be a vector function. It may not be a one-to-one map. However, we assume that there exists a function $\overline{f}_{\mathbf{h}}(\mathbf{u})$ defined on Ω such that $f(\mathbf{x}) = \overline{f}_{\mathbf{h}}(\mathbf{h}(\mathbf{x}))$ holds.

We assume that we can find $\mathbf{h}$ so that the function $\overline{f}_{\mathbf{h}}(\mathbf{u})$ is a concave function of $\mathbf{u}$ on Ω. Under this assumption, we can rewrite the regularization function $f(\mathbf{x})$ as:

$$f(\mathbf{x}) = \inf_{\mathbf{v}\in\mathbb{R}^n} \left[\mathbf{v}^T\mathbf{h}(\mathbf{x}) - f^*_{\mathbf{h}}(\mathbf{v})\right] \tag{2}$$

using concave duality (Page 308 in [2]). In this case, the function $f^*_{\mathbf{h}}(\mathbf{v})$ given below is the *concave dual* of $\overline{f}_{\mathbf{h}}(\mathbf{u})$:

$$f^*_{\mathbf{h}}(\mathbf{v}) = \inf_{\mathbf{u}\in\Omega} \left[\mathbf{v}^T\mathbf{u} - \overline{f}_{\mathbf{h}}(\mathbf{u})\right] \tag{3}$$

Moreover, it is well-known that the minimum of the right hand side of Eq. (2) is achieved at

$$\hat{\mathbf{v}} = \nabla_{\mathbf{u}}\overline{f}_{\mathbf{h}}(\mathbf{u})|_{\mathbf{u}=\mathbf{h}(\mathbf{x})} \tag{4}$$

This is a general framework. As $f(\mathbf{x}) = \alpha||\mathbf{x}||_p + (1-\alpha)||\mathbf{x}||_2$ for some $p \in (0,1)$, given any $h \geqslant 2$, Eq. (2) holds with $\mathbf{h}(\mathbf{x}) = \left[|x_1|^h, \ldots, |x_n|^h\right]$. The solution in (4) is given by,

$$\hat{v}_i = \frac{\alpha}{h}(\sum_j |x_j|^p)^{\frac{1}{p}-1}|x_i|^{p-h} + \frac{1-\alpha}{h}(\sum_j x_j^2)^{\frac{1}{2}-1}|x_i|^{2-h} \tag{5}$$

S3.2 Multi-stage convex relaxation

The solution of our tensor formulation is a stationary point of the following regularized optimization problem:

$$[\hat{\mathbf{x}}, \hat{\mathbf{y}}] = \arg\max_{\mathbf{x}\in\mathbb{R}^n, \mathbf{y}\in\mathbb{R}^m} \left[\frac{1}{2}\sum_{i,j,k} a_{ijk}x_ix_jy_k - \lambda f(\mathbf{x}) - \mu g(\mathbf{y})\right] \tag{6}$$

where $\lambda > 0$ and $\mu > 0$ are Lagrange multipliers. Since $f(\mathbf{x})$ is non-convex and $g(\mathbf{y})$ is convex, we consider a numerical procedure for solving Eq. (6) with convex loss and non-convex regularization $f(\mathbf{x})$. Let $h(\mathbf{x}) = \sum_j \mathbf{h}_j(\mathbf{x})$ be a convex relaxation of $f(\mathbf{x})$ that dominates $f(\mathbf{x})$ (for example, the smallest convex upperbound, i.e., the inf over all convex upperbounds). A simple convex relaxation of Eq. (6) becomes

$$[\hat{\mathbf{x}}, \hat{\mathbf{y}}] = \arg\max_{\mathbf{x}\in\mathbb{R}^n, \mathbf{y}\in\mathbb{R}^m} \left[\frac{1}{2}\sum_{i,j,k} a_{ijk}x_ix_jy_k - \lambda\sum_{j=1}^n \mathbf{h}_j(\mathbf{x}) - \mu g(\mathbf{y})\right] \tag{7}$$

It is possible that this simple relaxation yields a solution that is not close to the solution of (6). However, if $\mathbf{h}$ satisfies the condition of Subsection S3.1, then it is possible to write $f(\mathbf{x})$ as Eq. (2). In this new representation, we can rewrite Eq. (6) as

Inputs: tensor $\mathcal{A} = (a_{ijk})_{n\times n\times m}$, initial values $\mathbf{x}^{(0)} \in \mathbb{R}^n$ and $\mathbf{y}^{(0)} \in \mathbb{R}^m$.
Outputs: the gene membership vector $\mathbf{x}$ and network membership vector $\mathbf{y}$
Initialize $\hat{v}_j = 1$.
Repeat the following two steps (a stage) until convergence:

- Step 1: let $[\widehat{\mathbf{x}}, \widehat{\mathbf{y}}] = \arg\max_{\mathbf{x}\in\mathbb{R}^n_+, \mathbf{y}\in\mathbb{R}^m_+} \left[\frac{1}{2}\sum a_{ijk}x_ix_jy_k - \lambda\hat{\mathbf{v}}^T h(\mathbf{x}) - \mu g(\mathbf{y})\right]$.
- Step 2: let $\widehat{\mathbf{v}} = \nabla_{\mathbf{u}}\overline{f}_h(\mathbf{u})|_{\mathbf{u}=h(\mathbf{x})}$.

Figure S2. Multi-stage convex relaxation method for the tensor-based problem.

$$[\hat{\mathbf{x}}, \hat{\mathbf{y}}, \hat{\mathbf{v}}] = \arg\max_{\mathbf{x},\mathbf{v},\mathbf{y}} \left[\frac{1}{2}\sum_{i,j,k} a_{ijk}x_ix_jy_k - \lambda\mathbf{v}^T\mathbf{h}(\mathbf{x}) + \lambda f^*_{\mathbf{h}}(\mathbf{v}) - \mu g(\mathbf{y})\right] \tag{8}$$

This is clearly equivalent to Eq. (6) because of Eq. (2). If we can find a good approximation of $\hat{\mathbf{v}}$ that improves upon the initial value of $\hat{\mathbf{v}} = [1, \ldots, 1]^T$, then the above formulation can lead to a refined problem in $\mathbf{x}$ that is a better relaxation than Eq. (7).

Our numerical procedure exploits the above fact, trying to improve the estimation of v_j over the initial choice of $v_j = 1$ in Eq. (8) using an iterative algorithm. This can be done by repeatedly applying the following two steps:

- First, optimize $\mathbf{x}$ and $\mathbf{y}$ with $\mathbf{v}$ fixed.
- Second, optimize $\mathbf{v}$ with $\mathbf{x}$ and $\mathbf{y}$ fixed. This problem has the closed form solution given by Eq. (4).

Figure S2 presents our two-stage protocol to solve the regularized form of our problem. The procedure can be regarded as a generalization of concave-convex programming [3], which takes $h(\mathbf{x}) = \mathbf{x}$. By repeatedly refining the parameters in $\mathbf{v}$, we can obtain better and better convex relaxations leading to a solution superior to that of the initial convex relaxation with $v_j = 1$. The initial values of $\mathbf{x}$ and $\mathbf{y}$ could be uniform, randomly chosen, or taken from prior knowledge. In practice, an appropriate solver for Step 1, the time complexity of MSCR is often linear with respect to the total number of edges in the tensor.

Let $\mathbf{h}(\mathbf{x}) = \left[|x_1|^h, \ldots, |x_n|^h\right]$ and $h = 2$. This function is practically effective as the small convex upperbound of $f(\mathbf{x})$. The problem in Step 1 of Figure S2 can be approximately implemented by the power method presented in Figure S3. However instead of tuning λ and μ, this is implicitly done by using the gradient projection scheme so that the constraints $f(\mathbf{x}) = 1$ and $g(\mathbf{y}) = 1$ are enforced after each iteration via normalization. The idea is to optimize $\mathbf{x}$ with $\mathbf{y}$ fixed, then normalize $\mathbf{x}$ such that $\mathbf{x}$ satisfies the constraint $f(\mathbf{x}) = 1$. Similarly, we optimize $\mathbf{y}$ with $\mathbf{x}$ fixed, then normalize $\mathbf{y}$ such that $\mathbf{y}$ satisfies the constraint $g(\mathbf{y}) = 1$. The initial values $\mathbf{x}^{(0)}$ and $\mathbf{y}^{(0)}$ can be the vector with all entries one, i.e., $\mathbf{1} = [1, \ldots, 1]^T$. As discussed in Section S3.1, the solution of Step 2 is given in Eq. (5) when $f(\mathbf{x})$ is relaxed to $\mathbf{h}(\mathbf{x})$.

Inputs: tensor $A = (a_{ijk})_{n\times n\times m}$, vector $\hat{\mathbf{v}} \in \mathbb{R}^n$, initial values $\mathbf{x}^{(0)} \in \mathbb{R}^n$ and $\mathbf{y}^{(0)} \in \mathbb{R}^m$
Outputs: $\hat{\mathbf{x}}$ and $\hat{\mathbf{y}}$
Initialize $\mathbf{x} = \mathbf{x}^{(0)}$, $\mathbf{y} = \mathbf{y}^{(0)}$.
Repeat the following two updates until convergence:

- Update $\mathbf{x}$: $x_i \leftarrow \left[\frac{x_i \sum_{j,k} a_{ijk} x_j y_k}{\hat{v}_i}\right]^{\frac{1}{h}}$, then $\mathbf{x}$ is normalized by $x_i \leftarrow \frac{x_i}{\alpha||\mathbf{x}||_p + (1-\alpha)||\mathbf{x}||_2}$.
- Update $\mathbf{y}$: $y_k \leftarrow (y_k \sum_{i,j} a_{ijk} x_i x_j)^{\frac{1}{q}}$, then $\mathbf{y}$ is normalized by $y_k \leftarrow \frac{y_k}{||\mathbf{y}||_q}$.

Figure S3. Power method for updating **x** and **y** in Step 1 of the MSCR method shown in Figure S2

p \ α	0.2	0.4	0.6	0.8
0.2	1%	1%	1%	1%
0.4	31%	29%	28%	27%
0.6	100%	100%	100%	100%
0.8	100%	100%	100%	100%

Table S1. Hits table of performing our tensor method with different p and α on all 100 sets of networks, where hit $= \frac{\text{number of predefined patterns obtained/hit by tensor method}}{\text{number of all predefined patterns}}$. In this table, we chose the $\alpha = 0.2$ whose hitting values are always $\geqslant$ those of other α given the same p; similarly, we chose the $p = 0.8$ whose hitting values are always $\geqslant$ those of other p given the same α. They are highlighted by graying the first column and the last row.

S4 Simulation study

We generated 100 sets of random weighted networks with 300 genes, where each set contains 50 networks and 300 genes, and all their edges weights follow the uniform distribution in the range $[0, 1]$. Then a random RHS patterns with K_1 member genes and K_2 member networks are generated by making their edges weights follow the uniform distribution in the range $[\theta, 1]$. Here, K_1 is any of the four predefined values $\{6, 12, 18, 24\}$, K_2 is any of the five predefined values $\{5, 10, 15, 20, 25\}$, and θ is any of the five predefined values $\{0.5, 0.6, 0.7, 0.8.0.9\}$. Therefore, there are the total $4 \times 5 \times 5 = 100$ RHS patterns generated. Each RHS pattern is then placed into a set of networks, by randomly selecting K_1/K_2 genes/networks and replacing edges weights with the corresponding RHS pattern's edges weights. These simulated networks and patterns have taken into account of various factors that may affect the performance. We can evaluate the performance by counting the number of these predefined patterns found/hitted by the method with each parameter combination.

We performed our tensor method with different values of the parameters p and α on each set of networks and obtained the result as shown in Table S1. As explained in this table, $p = 0.8$ and $\alpha = 0.2$ is one of the best choices.

S5 The edge sampling procedure and its theoretical analysis

S5.1 Sampling procedure

Even though the MSCR method is efficient, its computation time can still be long for large sets of networks with many edges. In such cases, edge sampling can provide an efficient approximation to many graph problems [4, 5]. From the perspective of matrix or tensor computation, such sampling methods can be also viewed as matrix/tensor sparsification [6]. As RHS patterns predominately contain edges with large weights, we designed a non-uniform sampling method that preferentially selects edges with large weights. Specifically, each edge a_{ijk} is sampled with probability p_{ijk}:

$$p_{ijk} = \begin{cases} 1, & \text{if } a_{ijk} \geqslant \tilde{a} \\ p\left(\frac{a_{ijk}}{\tilde{a}}\right)^b, & \text{if } a_{ijk} < \tilde{a} \end{cases} \tag{9}$$

where $\tilde{a} \in (0,1)$, $b \in [1,\infty)$ and $p \in (0, \tilde{a}^b]$ are constants that control the number of sampled edges. Note that Eq. (9) *always* samples edges with weights $\geqslant \tilde{a}$. It selects an edge of weight $a_{ijk} < \tilde{a}$ with probability p_{ijk} proportional to the bth power of the weight. We choose $\tilde{a} = 0.6$, $b = 4$ and $p = 0.1$ as a reasonable tradeoff between computational efficiency and the quality of the sampled tensor, meanwhile satisfying the conditions of Theorem S5.1, i.e. $p \leqslant \tilde{a}^{b-1}$ and $b \geqslant 1$.

Procedure SAMPLING$(\mathcal{A}, p, \tilde{a}, b)$
for each $i \in [1,n], j \in [1,n], k \in [1,m]$ **do**
if $a_{ijk} \geqslant \tilde{a}$ **then**
$\widehat{a}_{ijk} = a_{ijk}$
else
$$\widehat{a}_{ijk} = \begin{cases} \min(\frac{a_{ijk}}{p_{ijk}}, \tilde{a}), & \text{with probability } p_{ijk} = p\left(\frac{a_{ijk}}{\tilde{a}}\right)^b \\ 0 & \text{with probability } 1 - p_{ijk} \end{cases}$$
return $\widehat{\mathcal{A}} = (\widehat{a}_{ijk})_{n\times n\times m}$

Figure S4. Edge sampling procedure of the tensor $A = (a_{ijk})_{n\times n\times m}$.

To correct the bias caused by this sampling method, the weight of each edge is corrected by its relative probability: $\widehat{a}_{ijk} = a_{ijk}/p_{ijk}$. The expected weight of the sampled network, $E(\widehat{a}_{ijk})$, is therefore equal to the weight of the original network. However, in practice, when the adjusted edge weight $\widehat{a}_{ijk} > \tilde{a}$ (but the original edge weight $a_{ijk} < \tilde{a}$), we enforced it to be $\widehat{a}_{ijk} = \tilde{a}$ for avoiding too large edge weights. The overall edge sampling procedure adopts the simple random-sampling based single-pass sparsification procedure introduced in [6]. Details of the sampling procedure is given in Figure S4. This single-pass sampling procedure's time complexity is $O(n^2m)$. It is obvious that the sparsification procedure in [6] is a special case of our sampling procedure when all entries of $\mathcal{A}$ are non-negative and $p = 1, \tilde{a} = \frac{\epsilon}{n+n+m}, b = 1$.

S5.2 Theoretical analysis

Based on the well-known Chernoff-Hoeffding bounds [7], we gave the bound of the non-zero entries in the corrected tensor $\widehat{\mathcal{A}}$ after sampling in Theorem S5.1. Therefore, the computational complexity

of the tensor MSCR algorithm on the tensor $\widehat{\mathcal{A}}$ after sampling is linear to the number of the non-zero entries of $\widehat{\mathcal{A}}$, i.e., $O(\frac{S}{\tilde{a}})$, with the probability at least $1-\exp(-\Omega(S'))$, where $S=\sum_{i,j,k} a_{ijk}$ and $S'=\frac{p}{\tilde{a}^b}\sum_{i,j,k} a_{ijk}^b$.

Theorem S5.1. *Given $p \leqslant \tilde{a}^{b-1}$, $b \geqslant 1$ and $0 \leqslant a_{ijk} \leqslant 1$ (for any i,j,k), with probability at least $1-\exp(-\Omega(S'))$, the tensor $\widehat{\mathcal{A}}$ contains at most $O(\frac{S}{\tilde{a}})$ non-zero entries, where $S=\sum_{i,j,k} a_{ijk}$ and $S'=\frac{p}{\tilde{a}^b}\sum_{i,j,k} a_{ijk}^b$.*

Proof. This proof is similar to Lemma 1 in [6]. Since $S=\sum_{i,j,k} a_{ijk}$, the number of a_{ijk} that are no less than $\tilde{a}$ is at most $\frac{S}{\tilde{a}}$; otherwise, the sum of all entries $\geqslant \tilde{a}$ would be greater than S. Now consider all non-zero a_{ijk} entries that are smaller than $\tilde{a}$.

The Chernoff bound [7] asserts that if $X_1, X_2, \ldots, X_N$ are indicator random variables and $X=\sum_i X_i$ with $\mathbb{E}[X]=\mu$, then for any $\delta > 0$

$$\mathbf{Pr}[X > (1+\delta)\mu] < \left(\frac{e^{\delta}}{(1+\delta)^{1+\delta}}\right)^{\mu} \tag{10}$$

In our case, we set up indicator random variables X_{ijk} which are 0 or 1 depending on whether $\tilde{a}=0$ or not. Then $X=\sum_{i,j,k} X_{ijk}$ is the number of non-zero entries of $\widehat{\mathcal{A}}$, and

$$\mu = \mathbb{E}[X] = \sum_{i,j,k} p_{ijk} = \frac{p}{\tilde{a}^b}\sum_{i,j,k} a_{ijk}^b = S' \tag{11}$$

Since $p \leqslant \tilde{a}^{b-1}$ and $a_{ijk}^b \leqslant a_{ijk}$ (because $b \geqslant 1$ and $0 \leqslant a_{ijk} \leqslant 1$), we have $\frac{p}{\tilde{a}^b}\sum_{i,j,k} a_{ijk}^b \leqslant \frac{1}{\tilde{a}}\sum_{i,j,k} a_{ijk}$. Then, we can get

$$\mu = \frac{p}{\tilde{a}^b}\sum_{i,j,k} a_{ijk}^b \leqslant \frac{1}{\tilde{a}}\sum_{i,j,k} a_{ijk} = \frac{S}{\tilde{a}} \tag{12}$$

Then we have

$$\mathbf{Pr}[X \leqslant (1+\delta)\frac{S}{\tilde{a}}] \quad \geqslant \quad \mathbf{Pr}[X \leqslant (1+\delta)\mu] \quad \geqslant \quad 1-\left(\frac{e^{\delta}}{(1+\delta)^{1+\delta}}\right)^{\mu} \tag{13}$$

We use the Chernoff bound with $(1+\delta)=e$ and arrive at the following

$$\mathbf{Pr}[X \leqslant e\frac{S}{\tilde{a}}] \quad \geqslant \quad 1-\exp(-\mu) = 1-\exp(-S') \tag{14}$$

So the claim holds.

□

S6 Details of computing platform and running time

Since the proposed tensor method is sequential, it can be performed on only one CPU processor. The computing platform was performed in a CPU processor of the computing node in the USC HPCC (High Performance Computing and Communications, website is `http://www.usc.edu/hpcc/systems/use-1-0/`) computing resources. This computing node consists of "Dual Quadcore Intel Xeon 2.5 GHz" and 12GB memory, and Linux OS. The memory used is about 10GB, and the running time is about

200 hours. To make the tensor method faster, we can sample less edges with the compromise of accuracy, because the time complexity of the tensor method is proportional to the number of non-zero entries in the tensor.

Since the proposed tensor method identifies RHSs sequentially one by one, it has the potential to be parallelized by simultaneously identifying multiple RHSs on multiple CPU processors. In the future work, we will develop the parallel version of the tensor method.

S7 Details of normalization procedure of pairwise gene co-expression correlations

We first compute the expression correlation between two genes as the minimum value of leave-one-out Pearson correlation coefficient estimates [8]. The resulting correlation estimate is conservative and sensitive to similarities in the expression patterns, yet robust to single experimental outliers. To make the correlation estimates comparable across datasets, we then applied Fisher's transform [9]. Given a correlation estimate r, Fisher's transformation score is calculated as $z = 0.5 \ln\left(\frac{1+r}{1-r}\right)$. Because we observed the distributions of z-scores to vary from dataset to dataset, we standardized the z-scores to enforce zero mean and unit variance in each dataset [10]. Then, the standardized correlations r' are obtained by inverting the z-score. Finally, the absolute value of r' is used as the edge weight of co-expression networks. Details of this procedure is given in Figure S5.

Input: expression profiles of n genes.
Output: estimated pairwise gene correlations r'_{ij} for any pair of genes i and j.

Compute correlation r_{ij} of each pair of genes i and j, by firstly computing their leave-one-out Pearson correlation coefficient estimates, then using the estimate whose absolute value is the minimum among absolute values of all estimates as the correlation r_{ij} of these two genes.

Normalize r_{ij} for any $1 \leqslant i, j \leqslant n$ with the following steps:

1. Apply Fisher's z transformation to r_{ij}, i.e., $z_{ij} = 0.5 \ln\left(\frac{1+r_{ij}}{1-r_{ij}}\right)$.
2. Standardize z_{ij}, i.e., $z'_{ij} = \frac{z_{ij}-\mu}{\sigma}$, where μ and σ are the mean and standard deviation of z_{ij} for all $1 \leqslant i, j \leqslant n$.
3. Apply Fisher's inverse transformation to z'_{ij}, i.e., $r'_{ij} = \frac{\exp(2z'_{ij})-1}{\exp(2z'_{ij})+1}$.

Return r'_{ij} for any i, j.

Figure S5. Procedure of estimating correlations of pairwise genes.

S8 Random network generation and comparison with real networks

To further evaluate the significance of RHSs discovered in real networks, we applied the proposed method on random networks to obtain RHSs. The random network is generated by randomizing a given network $G(V, E, W)$ (where V is the vertex set, E is the edge set and W is the sequence of the edges' weights corresponding to edges in E) with the following steps.

Given a real weighted network, the corresponding random weighted network is generated by a random redistribution of the actual weights on the randomly generated unweighted graph. In another words, for a real weighted network, we firstly generated a random unweighted network by using the widely used degree-preserving randomization procedure [11] (the MATLAB code is provided by the website `http://www.cmth.bnl.gov/∼maslov/matlab.htm`). Then the weights collected from the real weighted network were randomly distributed among the edges in the random unweighted network. This procedure is similar to our previous work [12] and is formally presented in Figure S6.

> **Procedure** GENERATE-RANDOM-NETWORK($G(V, E, W)$)
>
> **Generate random unweighted network:** apply the degree-preserving randomization procedure [11] on the edge set E to obtain a randomized edge set E' whose nodes' degree distribution is the same as that of E.
>
> **Randomly assign weights to edges of the random unweighted network:** randomly permutate the edge weights of the sequence W, and assign the values of the new randomized sequence W' to each edge of the edge set E'.
>
> **Return** the random weighted network $G'(V, E', W')$

Figure S6. Random weighted network generation procedure.

For our case, we applied this randomization procedure to each weighted network in the sparsified tensor $\widehat{\mathcal{A}}$. The resulted random tensor is denoted as $\widehat{\mathcal{A}}'$. We generated 100 random tensors $\widehat{\mathcal{A}}'$ from the same real tensor $\widehat{\mathcal{A}}$, and applied the proposed RHSs discovery method on each of them to discover the RHSs with $\geqslant 5$ genes, $\geqslant 5$ networks and "heaviness" $\geqslant 0.4$. Among 100 random tensors, None of RHSs were identified in any of the 100 times. When the minimum recurrence is 4 and other other remain unchanged, only 3 RHSs were found in all 100 random tensors. In contrast, 11,394 RHSs (and 4,327 representative RHSs) were identified in the real tensor. This comparison indicates the significance of RHSs identified in the tensor of real weighted networks.

S9 Signal extraction procedure of ENCODE data

Recently, in the ENCODE production phase (September 2007 $\sim$ present), there are 191 ENCODE genome-wide tables for ChIP-seq. To perform enrichment analysis, we integrated these ChIP-seq samples in Genome-wide ENCODE data including chromatin modification, TF binding, Methylation, and chromatin accessibility. UCSC database provide the peak tables for these ChIP-Seq data.

However, different data types have different criterion for the significance. We used both top N and threshold condition to select peaks as follows:

1. If the table is Methylation-Seq data, select the peaks score > 0.6;
2. Else if the peaks have p-value, select the peaks with p-value < 1E-4 and top 20K;
3. Otherwise, select the peaks with signal value > 1 and top 20K.

Then for each table, we selected the target genes whose transcriptional start site is nearby the peaks within 5000 bases.

S10 Comparisons with unweighted networks

The weighted networks were transformed to unweighted networks by dichotomizing edges with an expression correlation cutoff of 0.6. The proposed tensor method was then applied to both weighted and unweighted networks. We compared rates of protein complex/pathway/transcriptional factor homogeneity detected in the top $K = 200, 400, \cdots, 2000$ modules, ranked by recurrences or average heaviness in their datasets of occurrence. Figure S7 and S8 demonstrated that weighted graph analysis consistently outperforms unweighted graph analysis

S11 Detailed information of modules in the regulatory network reconstructed in Figure 9 of the manuscript

Please see Table S2 for details.

Table S2. Member genes of RHSs/modules in the regulatory network reconstructed on the basis of the derived transcription networks in Figure 10 of the manuscript.

from representative RHS/module	member genes of the module
M1307	RPL37, RPS25, RPL17, RPL31, RPS8, RPS20, RPS27, RPS6, RPS15A, SRP14, RPS7, RPS23, RPS17
M1640	RPL17, RPS6, RPS3A, RPL30, PHB2, RPS7, RPL11, PFDN5, GDI2, UQCRQ, SNRPD2, NDUFA1
M823	RPS27, RPS17, GAPDH, RPS29, RPS16
M1222	RUVBL2, MCM2, CCNB2, MCM5, DDX39
M143	RPL3, RPL9, RPS3A, RPS2, RPS12, RPL8, RPL11, RPL7, RPL12, RPS10, RPS4X, RPS8, FAU, RPL31, RPS21, RPL10A
M2140	RPL39, RPS20, RPL27, RPS23, RPL13
M2075	PHB2, RPS8, EEF2, RPS2, RPL19, RPL27, RPL17, RPS15, RPL11, RPS17, RPS21, FAU, RPL30, RPL29, RPS20
M10	RPS3A, EEF1A1, RPL41, TPT1, RPS27, RPS29, RPL9, RPS4X, RPS12, RPS13, RPL7, RPS6, RPS15A, FAM131B, CYP2C18, PPP2R5E

Continued on next page

Table S2 – continued from previous page

from representative RHS/module	member genes of the module
M3295	MDH1, HAX1, NDUFB1, NDUFAB1, UQCRQ
M3768	NDUFB5, COX7C, ATP5H, ATP5L, PARK7
M1301	HMMR, KIF11, TTK, KIAA0101, ZWINT, RRM1, DLGAP5, H2AFZ, KIF2C, GINS1, KIF14, CYP2C18, PPP2R5E, FAM131B, ZPBP
M3332	CKS2, TPX2, SNRPD1, PTTG1, CCNB1
M1838	RPL13, RPS19, RPL4, RPS9, RPS16
M2524	MAD2L1, MCM6, HMGB2, HSPD1, RBMX
M564	RPS9, RPS15, RPL19, RPL27, RPS21, RPS23, RPS2, RPS27, RPS13, RPS8
M867	RPS8, RPL7, RPL30, RPS25, RPL11, RPS27A, RPS6, RPL9, EIF3H, RPL10A, RPL27, RPS3A, RPL35
M1294	RPL17, RPL12, RPS23, PPIA, OAZ1
M20	PSMD14, PSMA3, VBP1, PSMA4, SNRPG, H2AFZ, HAT1, COPS5, RAN, MRPL3, C11orf58, SRP9, HNRNPA2B1
M1712	RPL10A, RPL29, PFDN5, RPL12, RPS19, RPS10, RPL11, RPL8, FAU, RPS12
M1661	CHAF1A, SPAG5, MCM3, MCM4, HMMR, CDC6, MCM7, FEN1, FANCI, KIFC1, NCAPD2, LMNB2, WHSC1, LMNB1, FAM131B, CYP2C18
M1472	RPL19, RPS12, RPL10A, RPS20, RPS29, UBB, RPS6, RPL27, RPS27, RPS17
M932	RPS13, RPS15A, RPS6, RPL12, RPL11
M1585	CKS2, TRIP13, MKI67, FOXM1, BIRC5, KIF2C, AURKB, ESPL1, STMN1, TK1, PPP2R5E, ZPBP, CYP2C18
M441	RPL29, RPS3A, RPL11, RPS8, PFDN5, RPL31, RPS10, RPL3, RPS6, RPS17, RPS7, RPL10A, NACA, RPL17, RPL9
M32	MAD2L1, H2AFZ, RRM1, HMGB2, MCM6, DEK, HAT1, SFRS3, PPP2R5E
M1731	ATP5L, RPS21, FAU, RPS29, RPS13, COX7A2, RPS25, ATP5I, RPS12, RPS17, RPS27A, EIF3F, RPL31, RPS23, RPS10, RPS15A, EIF3H, RPS27
M2001	RPL10, GNB2L1, RPS20, RPS6, RPS4X, RPL9, GNAS, PPP2R5E
M3676	COX7B, DBI, CYCS, COX6C, HSPE1
M1666	RPL13, PTMA, RPS2, RPL19, RPS16
M1464	RPS11, RPS15A, RPL29, RPL10A, RPS27
M2589	NASP, SMC4, POLD1, MCM2, HPRT1
M1739	RPLP1, RPL17, RPL13, RPS15, RPL4
M1604	BUB1, CENPE, PLK4, CENPF, GTSE1, KIF11, POLD1, PTTG1, FANCI, KIF23, NCAPD2, AURKA, MYBL2, KIFC1, CENPA, FAM131B
M709	ILF3, MCM7, SSRP1, NONO, PTBP1, MCM3, MCM2, PPP2R5E
M1468	RPS9, RPS21, RPL10A, RPS19, RPL27
M1121	RPS12, RPL4, RPS13, EIF3E, RPS20
M3718	NDUFB5, NDUFB6, ATP5J2, COX7B, NDUFB3

Continued on next page

Table S2 – continued from previous page

from representative RHS/module	member genes of the module
M426	RPS7, HNRNPA1, NACA, SNRPE, RPL17, TOMM20, RBMX, EIF3H, EIF3E, UBA2
M115	RPS20, RPL30, RPS27, RPL11, RPS13, RPS12, FAU, RPL31, RPS8, RPS21, RPL24, RPL9, RPS4X, RPS3A, RPS16
M1084	RPL27, RPS13, NPM1, RPL30, RPS25, RPS24
M1617	RUVBL2, CKS1B, TRIP13, TUBB2C, SNRPA, KIF2C, MKI67
M3308	TRIM28, FBL, SNRPB, SNRPA, FARSA
M898	COX6B1, NDUFAB1, UQCRQ, COX8A, ATP5L, COX7A2
M1137	BTF3, COX7C, RPL11, RPL7, RPL10A, RPS7, RPL9, RPS3A, COX7A2L, RPL24

References

[1] Zhang T (2010) Analysis of multi-stage convex relaxation for sparse regularization. J Mach Learn Res 11: 1081–1107.

[2] Rockafellar RT (1970) Convex Analysis. Princeton, NJ: Princeton University Press.

[3] Yuille AL, Rangarajan A (2003) The concave-convex procedure. Neural Comput 15: 915–936.

[4] Tsay AA, Lovejoy WS, Karger DR (1999) Random sampling in cut, flow, and network design problems. Math of Oper Res 24: 383–413.

[5] Achlioptas D, McSherry F (2007) Fast computation of low-rank matrix approximations. J ACM 54.

[6] Arora S, Hazan E, Kale S (2006) Approximation, Randomization, and Combinatorial Optimization. Algorithms and Techniques, Berlin Heidelberg: Springer-Verlag, chapter A Fast Random Sampling Algorithm for Sparsifying Matrices. pp. 272–279.

[7] Motwani R, Raghavan P (1995) Randomized Algorithms. Cambridge University Press.

[8] Zhou X, Kao M, Huang H, Wong A, Nunez-Iglesias J, et al. (2005) Functional annotation and network reconstruction through cross-platform integration of microarray data. Nat Biotechnol 23: 238–243.

[9] Anderson TW (2003) An introduction to multivariate statistical analysis. Hoboken, NJ: Wiley-Interscience, 3 edition.

[10] Xu M, Kao MCJ, Nunez-Iglesias J, Nevins JR, West M, et al. (2008) An integrative approach to characterize disease-specific pathways and their coordination: A case study in cancer. BMC Genomics 9: S12.

[11] Maslov S, Sneppen K (2002) Specificity and stability in topology of protein networks. Science 296: 910–913.

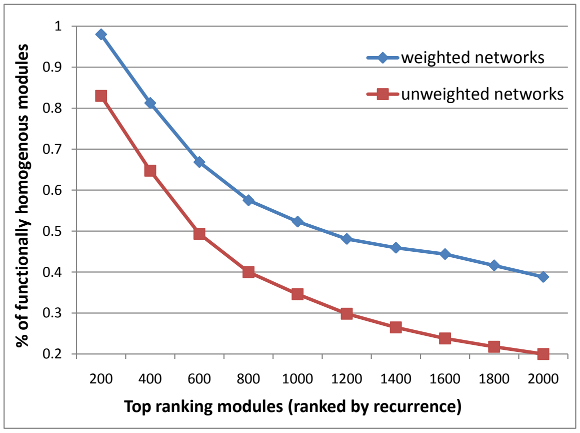

(A) protein complex homogeneity and rank by their recurrences

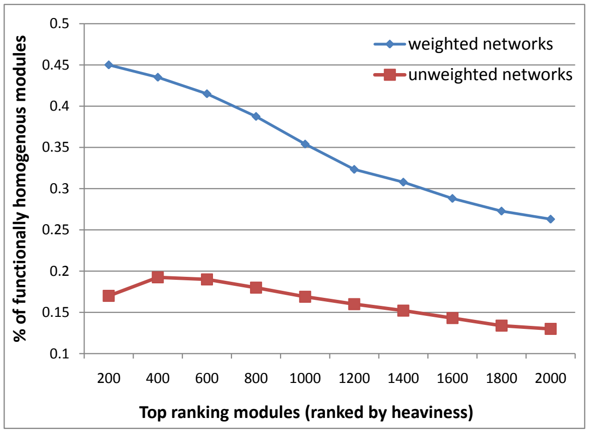

(B) protein complex homogeneity and rank by their average heaviness in active datasets

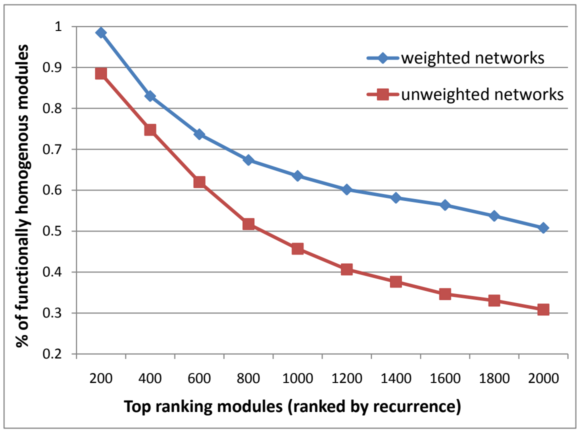

(C) pathway homogeneity and rank by their recurrences

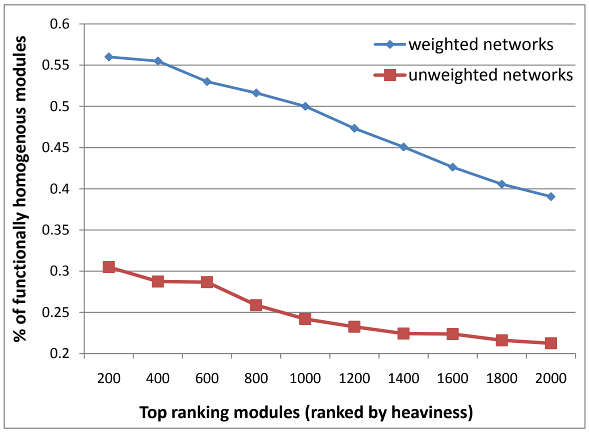

(D) pathway homogeneity and rank by their average heaviness in active datasets

Figure S7. Comparison between weighted and unweighted network analysis in terms of protein complex and pathway homogeneity.

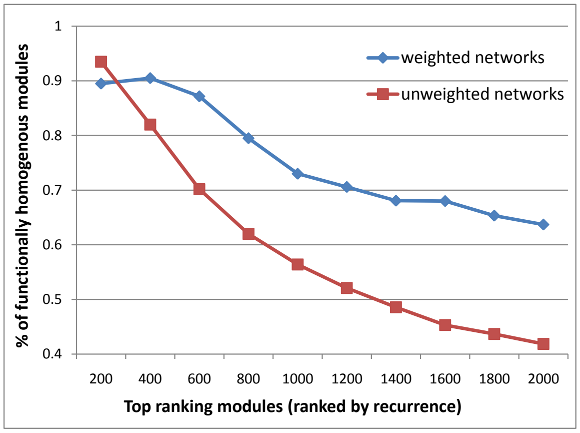

(A) transcriptional (from ENCODE data) homogeneity and rank by their recurrences

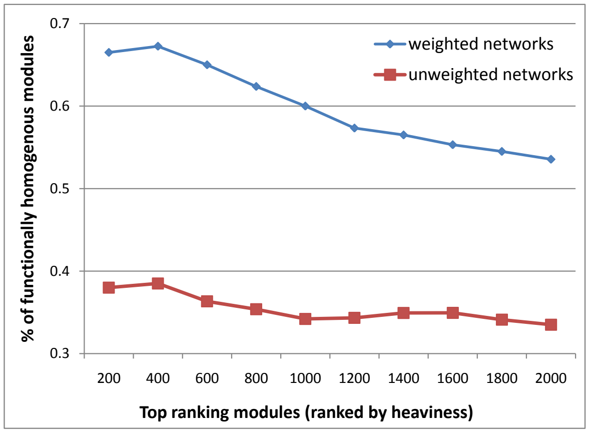

(B) transcriptional (from ENCODE data) homogeneity and rank by their average heaviness in active datasets

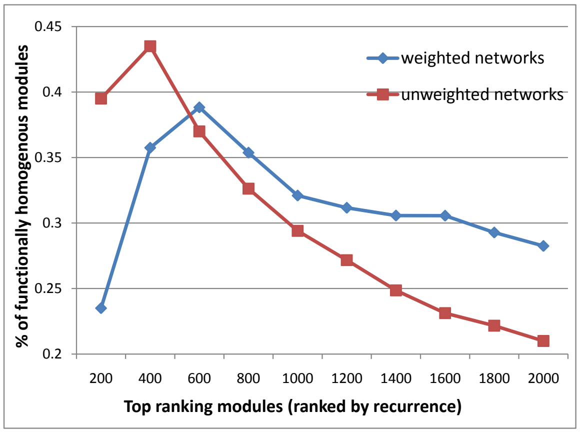

(C) transcriptional (from ChIP-chip data) homogeneity and rank by their recurrences

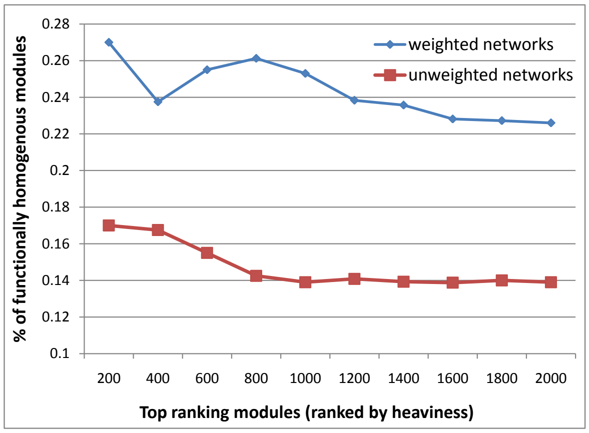

(D) transcriptional (from ChIP-chip data) homogeneity and rank by their average heaviness in active datasets

Figure S8. Comparison between weighted and unweighted network analysis in terms of transcriptional homogeneity.

[12] Li W, Lin Y, Liu Y (2007) The structure of weighted small-world network. Physica A 376: 708–718.
